# Study and analysis of antitumor resistance mechanism of PD1/PD‐L1 immune checkpoint blocker

**DOI:** 10.1002/cam4.3410

**Published:** 2020-09-02

**Authors:** Zhengyi Wang, Xiaoying Wu

**Affiliations:** ^1^ GCP Center of Sichuan Academy of Medical Sciences and Sichuan Provincial People’s Hospital Medical Sciences Chengdu City Sichuan Province China; ^2^ Institute of Laboratory Animals of Sichuan Academy of Medical Sciences and Sichuan Provincial People’s Hospital Chengdu City Sichuan Province China; ^3^ Ministry of Education and Training Second People’s Hospital Chengdu City Sichuan Province China

**Keywords:** immunocheckpoint blocker, mechanism, PD‐1/PD‐L1, resistance, study and analysis

## Abstract

Immunocheckpoint proteins of tumor infiltrating lymphocytes play an important role in tumor prognosis in the course of tumor clinicopathology. PD‐1 (Programmed cell death protein 1) is an important immunosuppressive molecule. By binding to PD‐L1 (programmed cell death‐ligand 1), it blocks TCR and its costimulus signal transduction, inhibits the activation and proliferation of T cells, depletes the function of effector T cells, and enables tumor cells to achieve immune escape. In recent years, immunocheckpoint blocking therapy targeting the PD‐1/PD‐L1 axis has achieved good results in a variety of malignant tumors, pushing tumor immunotherapy to a new milestone, such as anti‐PD‐1 monoclonal antibody Nivolumab, Pembrolizumab, and anti‐PD‐L1 monoclonal antibody Atezolizumab, which are considered as potential antitumor drugs. It was found in clinical use that some patients obtained long‐term efficacy, but most of them developed drug resistance recurrence in the later stage. The high incidence of drug resistance (including primary and acquired drug resistance) still cannot be ignored, which limited its clinical application and became a new problem in this field. Due to tumor heterogeneity, current limited research shows that PD‐1 or PD‐L1 monoclonal antibody drug resistance may be related to the following factors: mutation of tumor antigen and antigen presentation process, multiple immune checkpoint interactions, immune microenvironment changes dynamically, activation of oncogenic pathways, gene mutation and epigenetic changes of key proteins in tumors, tumor competitive metabolism, and accumulation of metabolites, etc, mechanisms of resistance are complex. Therefore, it is the most urgent task to further elucidate the mechanism of immune checkpoint inhibitor resistance, discover multitumor universal biomarkers, and develop new target agents to improve the response rate of immunotherapy in patients. In this study, the mechanism of anti‐PD‐1/PD‐L1 drug resistance in tumors, the potential biomarkers for predicting PD‐1 acquired resistance, and the recent development of combination therapy were reviewed one by one. It is believed that, based on the complex mechanism of drug resistance, it is of no clinical significance to simply search for and regulate drug resistance targets, and it may even produce drug resistance again soon. It is speculated that according to the possible tumor characteristics, three types of treatment methods should be combined to change the tumor microenvironment ecology and eliminate various heterogeneous tumor subsets, so as to reduce tumor drug resistance and improve long‐term clinical efficacy.

## Graphical abstract

Based on the analysis of existing studies, ICB resistance involves complex network regulatory mechanisms, and the countermeasures against certain drug resistance mechanisms cannot solve the drawbacks of clinical drug resistance. In terms of treatment, traditional basic treatment, evidence‐based treatment of tumor type, and individual precision therapy should be integrated to comprehensively remove all subsets of cells in heterogeneous tumors.

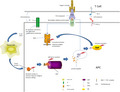

ICB (immune checkpoint blockers) act on T‐cell immunosuppressive targets such as CTLA‐4(Cytotoxic T lymphocyte‐associated antigen‐4), PD‐1(Programmed cell death protein 1), or block immunocheckpoint‐related ligands such as PD‐L1(Programmed cell death ligand 1), bringing hope to patients with refractory tumors,^1^ as an important means of tumor immunotherapy, it can significantly improve the prognosis of tumor patients. Studies have shown that after the interaction between PD‐L1 and PD‐1, phosphorylation of the ITIM (Immunoreceptor Tyrosine‐based Inhibitory Motif) and ITSM (Immunoreceptor Tyrosine‐based Switch Motif) of the latter can be induced. Subsequently, protein tyrosine phosphatase SHP‐2 (Src homology phosphatase 2) is recruited to weaken the activation signal of T cells and mediate immune escape. The application of ICB can block the recruitment of SHP‐2, lose the phosphatase dephosphorylation, and then activate T cells to play the immune function, as shown in Figure 1.

**FIGURE 1:**
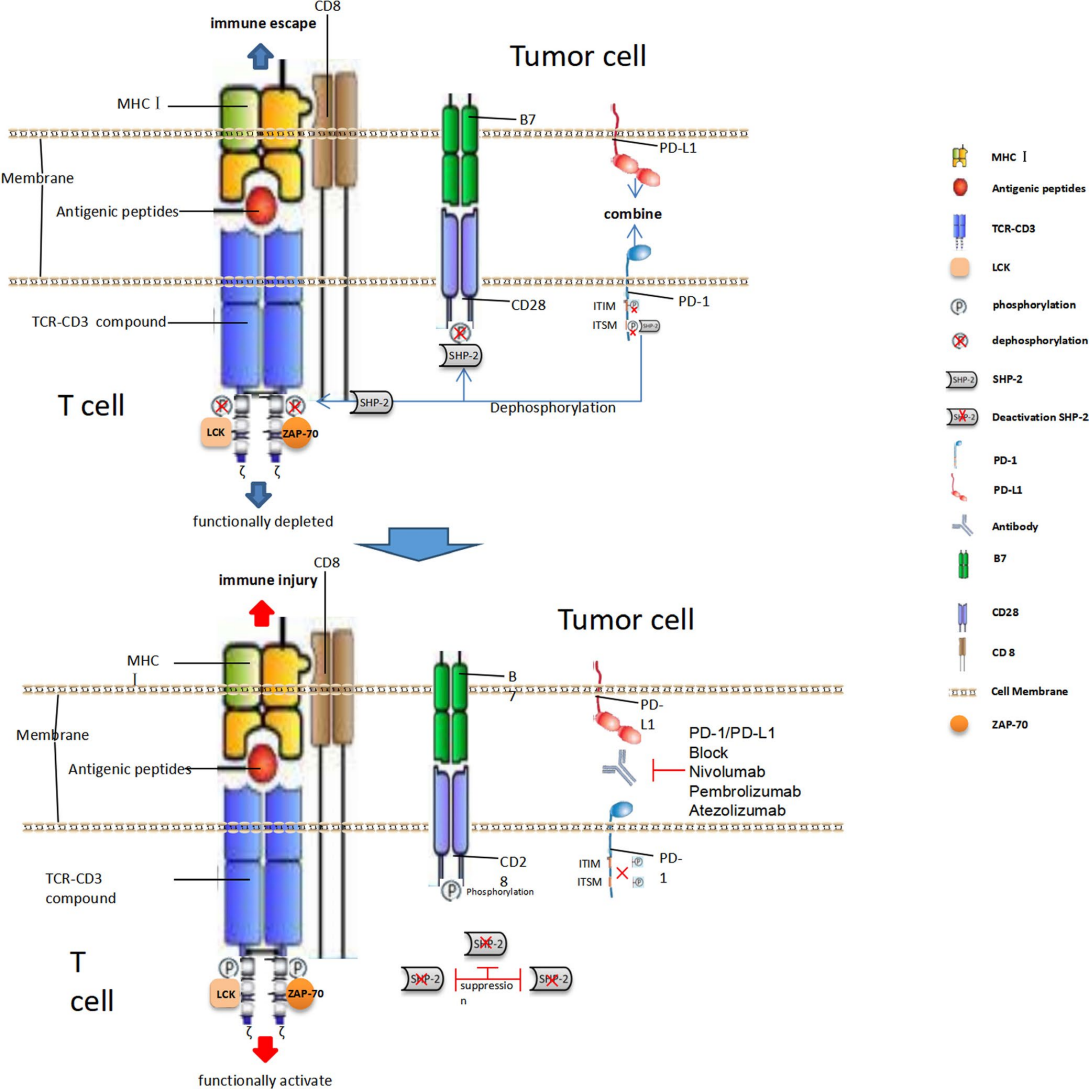
Molecular mechanism of PD‐1/PD‐L1 blocker action.PD‐1/PD‐L1. After PD‐1/PD‐L1 conjugation, the ITIM and ITSM structures in the PD‐1 cell membrane region were phosphorylated, and phosphatase SHP‐2 was recruited to the cell membrane region, so that the TCR and CD28 membrane domains were dephosphorylated, and the first signal and co‐stimulation signals of T cells were activated, which could not be transmitted to the downstream proteins, and the T cells could not be activated. When PD‐1 / PD‐L1 monoclonal antibody is activated, the intramembrane motif of PD‐1 cannot be phosphorylated, and SHP‐2 recruitment is lost. There is no phosphatase dephosphorylation, and the activation signals of TCR and CD28 can be transmitted to the downstream proteins, finally stimulating the proliferation and differentiation of T cells

However, clinical studies have found that although the PD‐1/PD‐L1 blocking therapy has achieved an unprecedented sustained response rate in a variety of malignant tumors, most patients have not benefited from the treatment, and some responders have relapsed after a period of response, that is, drug resistance has occurred. Some studies have found that the tumors significantly shrink or do not progress after PD‐1 blocking treatment, accounting for 48% of the total number of studies; after the treatment, the tumor directly increased or shrank first and then increased, accounting for 52% of the total number of patients.^2^ Other studies have suggested that remission rates rarely exceed 40% in most tumors, most of which are partial.^3^ Compared with molecular‐targeted drugs, immune checkpoint blocking tumor therapy has a higher incidence of drug resistance. Usually, treated patients, according to the benefit of immune checkpoint blockers can be classified into the following several kinds of people: (1) Effective: after medication can continue to control tumor progression; (2) The primary drug resistance: initial no drug treatment effect; and (3) The secondary resistance (acquired drug resistance): initial can relieve tumor progression, but late failure.^4^ The mechanism of primary and secondary ICB resistance may be related to heterogeneity of tumor formation process. In the process of tumor immunotherapy, although the time and degree of drug resistance are different in different drug‐resistant populations, there is no difference in essence, which is a means of tumor immune escape. Since it is not fully known what biological processes determine the results of effective or drug resistance, ICB tolerance may involve the interaction of various internal and external factors between immune cells and heterogeneous tumor cells within the tumor, and the tolerance mechanism may show a dynamic multilevel change process, so it has become the focus of current research on tumor treatment. Among the many drug resistance mechanisms, there have been many reports on the regulation of TME (tumor microenvironment), intracellular protein mutations, oncogene signal transduction pathways, epigenetic changes, and other related studies. However, in clinical practice, they only play a partial role in the improvement of tumor ICB resistance, and it is difficult to obtain universal biomarkers. In this study, the existing mechanism of ICB resistance was elaborated and analyzed one by one, in the hope of obtaining a more beneficial treatment idea for clinical efficacy.

## 1 EFFECT OF TUMOR‐ASSOCIATED ANTIGEN EXPRESSION AND ANTIGEN PRESENTING DYSFUNCTION ON CLINICAL DRUG RESISTANCE OF PD‐1/PD‐L1 MONOCLONAL ANTIBODY

Tumor cells are different from normal cells in that they are immunogenicity due to gene mutations that induce cells to express a series of neospecific antigen protein phenotypes. However, heterogeneity is one of the important characteristics of tumors. There are significant differences in the expression of cell surface antigens between different types of tumors or among subgroups within the same tumor, showing different levels of protein immunogenicity. One of the most direct reason for the failure of PD‐1/PD‐L1 antibody to treat tumors (primary or secondary drug resistance) is the lack of high immunogenicity tumor‐specific antigen, leading to the failure of T cells to recognize it. Neoantigen epitope protein sequence formed by tumor‐specific DNA mutation, which regulates the differentiation of TIL (tumor infiltrating lymphocytes), is a necessary condition for the effectiveness of PD‐1/PD‐L1 monoclonal antibody. Studies have shown that the more effective tumor‐specific antigens are formed, the better the efficacy of PD‐1/PD‐L1 blockers is, and it is related to the clinical PFS (progression‐free survival) of patients, such as highly immunogenic melanoma, renal cell carcinoma, and non‐small cell lung cancer. Most of them are sensitive to the clinical treatment of PD‐1/PD‐L1 antibodies. If tumor‐specific antigen expression is too low and immunogenicity is weak, it is not enough to activate the original T cells; if the specific antigen structure is similar to immune‐tolerant antigens or autoantigens, APCs (antigen presenting cells) cannot recognize it and cannot initiate T‐cell activation, which leads to drug resistance of PD‐1/PD‐L1 blockers, such as low‐immunogenic tumors, pancreatic cancer and prostate cancer, and poor response to PD‐1/PD‐L1 antibody treatment to a large extent.^5^, ^6^ T‐cell‐dependent immunoscreening, as one of the important mechanisms of tumor immune editing, can eliminate tumor cell subsets with high immunogenicity of mutant‐associated antigens under its pressure,^7^ or, based on immune editing pressure, tumor cells take an active approach and selectively “disappear” high‐immunogenicity–specific antigen subsets from tumors by gene expression reduction or mutant allele deletion.^8^, ^9^ The loss of mutation‐related tumor‐specific antigens during the treatment process can lead to acquired drug resistance. If both the initial tumor cells and the differentiated subgroups of tumor cells lack high‐immunogenicity–specific antigens and cannot activate tumor‐infiltrating T lymphocytes, primary drug resistance can be directly caused.

.In general, tumor cells use MHCI molecules to present tumor antigen peptides to the cell surface, which are phagocytosed and recognized by APC cells, and then present to CD8^+^, CD4^+^ T lymphocyte via MHCI and MHCII molecules of APC cells , respectively. Under the synergistic effect of the costimulus signaling molecules of the second signaling pathway CD28/B7 (CD86 and CD80), activate T cells, promote a large number of T cells to differentiate and proliferate, and form memory T lymphocytes. CTLs (Cytotoxic T lymphocytes) can recognize specific antigens on the surface of tumor cell membranes and secrete granulase and perforin, killing the binding tumor cells and completing the adaptive immune clearance of tumors. However, tumor by secrete IL‐10 (Interleukin 10), VEGF (Vascular endothelial growth factor), and so on inhibiting factor prompted the myeloid cells abnormal differentiation, APC‐related precursor DC (Dendritic cells) decrease, at the same time, peripheral immature DC due to no expression or low expression costimulatory molecules, CD80 and CD86 cannot be activated, thus, the number of normal mature DCs in peripheral blood decreases, while the number of immature DCs increases. When the tumor recruits these immature DCs, it cannot activate effector T cells when presenting the antigen, but can induce the production of Tregs(Regulatory T cells).^10^ Because PD‐1/PD‐L1 blockers cannot activate the initial T cells in the absence of antigen stimulation signal, the body does not produce an immune response at this time, so drug resistance and immune escape occur; such patients are not suitable for immunotherapy programs.

**FIGURE 2:**
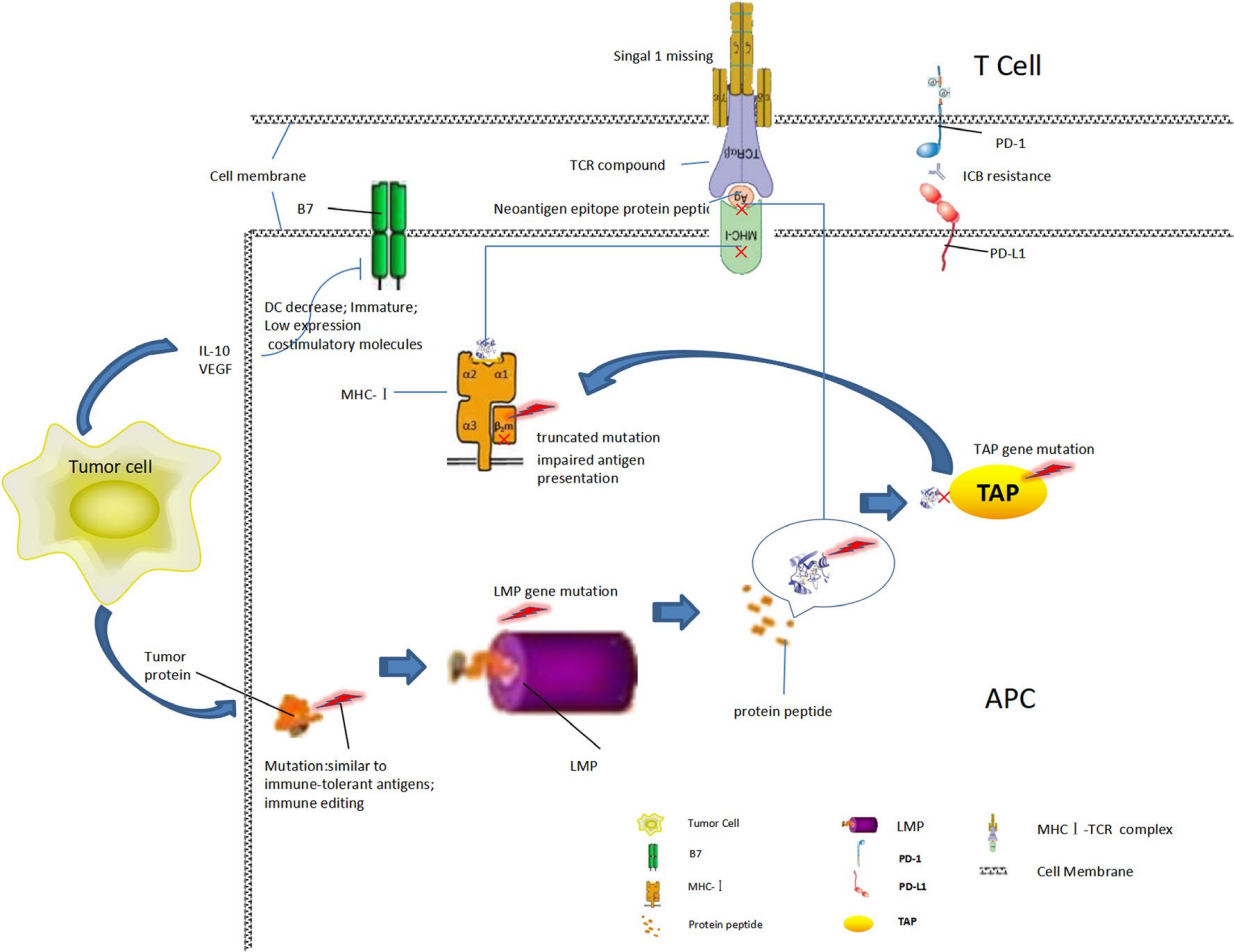
Resistance mechanism of immune PD‐1/PD‐L1 monoclonal antibody during antigen presentation.Various protein mutations result in the inability of specific antigen proteins to be processed and presented, and the loss of the first signal of effector T cells, which cannot be activated, causes AntiPD‐1/PD‐L1 tolerance

In addition, for within the cell, antigen processing, transport, presented protein molecules, such as MHC class I molecules, β2M (Beta 2 microglobulin), LMP (Large multifunctional protease), and the TAP (Transporter associated with antigen processing) is a tumor antigen processing and/or presented important component, when encoding their genetic change can also lead to the ICB resistance. For example, β2M is involved in the folding and transportation of MHCⅠ molecules, and its truncated mutation can lead to impaired expression of the latter on the surface of APCs, resulting in impaired antigen presentation and immunotherapy resistance.^11^ Abnormal mutation of β2M is considered to be an important mechanism of tumor resistance to T‐cell–mediated immune response, and also one of the causes of immunotherapy resistance.^12^ As shown in Figure 2. to sum up, in ICB immunotherapy, the degree or level of presentation of specific tumor antigenic epitope to T cells has a decisive influence on the therapeutic effect, and abnormalities in each link of inducing activation and producing effector T cells can induce ICB drug resistance. For example, the higher the TMB (Tumor gene mutation burden) is, the higher the expression and production of specific tumor antigen protein will be, and the increase in abnormal protein will promote the increase in APC presentation level, which is more likely to excite initial effector T cells and produce immune effect.

Among them, TMB level, APC efficacy, and molecular dysfunction related to various levels of protein delivery chains (including functional or structural) all contribute to drug resistance. Tumor microenvironment is the carrier for the occurrence of these abnormalities, these functions, or organic disorder, mostly in the pathology of the tumor cells build environment, two factors supplement each other (APC generation, eg, is influenced by cytokines secreted by tumors). It is suggested that in the search for the solution of abnormal antigen presentation IBC resistance, the eye should not be limited to a single protein or cell, but should be comprehensively analyzed through the whole antigen presentation effect chain, and eliminate all the pathological factors that are not beneficial to the effective presentation of the antigen, so as to obtain the best clinical effect. Therefore, this treatment is not a single type of drug treatment, but a combination of drugs should be used. In clinical diagnosis, biomarker detection should also focus on the whole reaction chain, such as phenotypic analysis of peripheral blood DC cells, functional test combined with TMB analysis, and various cytokine detection, which may become important predictors of clinical ICB treatment response.

## 2 EFFECT OF NON‐PD‐1/PD‐L1 INHIBITORY IMMUNOCHECKPOINT ACTIVATION ON CLINICAL DRUG RESISTANCE OF PD‐1/PD‐L1 MONOCLONAL ANTIBODY

Except the PD‐1, a variety of high expression of immune inhibitory checkpoints are associated with T‐cell function, such as TIM‐3 (T cells immune globulin mucin‐3), CTLA 4, LAG3 (Lymphocyte activation gene 3), BTLA (B and T lymphocytes attenuation factor), and TIGIT (T‐cell immune globulin and ITIM structure domain proteins), etc, and these checkpoints also have an impact on the efficacy of PD‐1/PD‐L1 antibody.^13^, ^14^, ^15^ TIM‐3 (*HAVCR2*) is an immunosuppressant checkpoint molecular protein expressed on the surface of activated T cells, NK cells, and monocytes. After binding to the ligand galectin‐9, effector cells lose function and go into apoptosis.^16^ The study found that in drug‐resistant tumors, the higher the binding degree of T cells with PD‐1 blocker, the stronger the expression of Tim‐3 in T cells, suggesting that TIM‐3 acquisition increased in T cells after drug resistance. Combined application of PD‐1/PD‐L1 antibody and TIM‐3 antibody can better inhibit tumor growth, suggesting that the upregulation of Tim‐3 expression may be involved in the acquired resistance of PD‐1 blocker.^17^ LAG3 is an immune checkpoint molecule expressed by activated T cells, NK cells, and B cells,^18^ binding to MHCⅡ and Galectin‐3 (Galactose lectin‐3), inhibiting effector T‐cell function, and enhancing Treg cell activity.^19^ TIGIT, as an emerging immune checkpoint receptor, can promote the depletion of T cells and inhibit the antitumor immune response mediated by T cells, thus, promoting tumor development. Recent studies have shown that TIGIT can not only promote T‐cell depletion but also mediate NK cell depletion, and activate potential antitumor memory response after blocking TIGIT.^20^ Mechanistically, these subsequent upregulation of inhibitory molecules is associated with activation of multiple cancer‐related pathways, such as PI3K(Phosphoinositide 3‐kinase)/AKT(Protein kinase B) and interferon signaling pathways.^21^, ^22^, ^23^, ^24^ Thus, when one immunocheckpoint（PD‐1/PD‐L1） is suppressed, other immunocheckpoint may be induced, and the combination of anti‐PD‐1/PD‐L1 and other co‐expressed immunocheckpoint blocking antibodies such as CTLA‐4 inhibitors^25^, ^26^ and LAG3 inhibitors^27^ can enhance the antitumor response in patients with severe T‐cell failure. The clinical effect is very promising, which is also one of the hot spots of immunotherapy research. In addition to some inhibitory checkpoints mentioned above, NRP‐1 (Neuiropilin‐1), 2B4 (Natural killer cell receptor 2B4), BTLA (B‐ and T‐lymphocyte attenuator), PDPN (Podoplanin), PROCR (Protein creceptor), CD160, etc also showed the same inhibitory immune checkpoint action, distributed on the surface of different types of immune cell membranes, and their corresponding ligands were distributed in various tumor tissues. It can be seen that the immune "brake" function of immune checkpoint is a cluster effect, each checkpoint function is complementary, the whole cooperation exerts the immunosuppressive effect. So, single antagonistic PD1/PDL1 pathway has limited function in improving immune cells and is prone to drug resistance. Therefore, in clinical treatment, the most direct strategy for the solution of non‐PD1/PD‐L1 checkpoint resistance is to combine multiple checkpoint inhibitors to alleviate their inhibition on immune effector cells and play the role of immune clearance. It is worth noting that in heterogeneous tumor tissues and their microenvironment, there are various immune checkpoint‐activated ligand molecules, which can effectively inhibit T effector cells locally and regulate tumor immune escape. Rational analysis and treatment of these ligand molecules in the treatment strategy determine the efficacy and prognosis of tumor immunotherapy, and the biomarker characteristics of these ligand molecules can be the basis for the combination regimen of immunocheckpoint inhibitors.

## 3 INFLUENCE OF TUMOR MICROENVIRONMENT ON CLINICAL TREATMENT RESISTANCE OF PD‐1/PD‐L1 MONOCLONAL ANTIBODY

Various cytokines, chemokines, chemical factors, secretory proteins, exocrine bodies, immune cells, fibroblasts, interstitial cells, and other interactions in TME together form a complex network that regulates tumor immunity and is also an important factor leading to resistance to PD‐1/PD‐L1 checkpoint blockers. TMEs is heterogeneous. Since different types of TMEs have different effects on the infiltration, distribution, and function of effector T cells and immunosuppressive cells in tumors, they can lead to different clinical effects of PD‐1/PD‐L1 blockers and are important reasons for ICB drug resistance. TMEs can be divided into several types according to the distribution of tumor inflammatory cell infiltration, PD‐L1 and CD8B gene expression level in tumor.^28^, ^29^ Usually can be divided into the following two types: infiltrated–excluded (I‐E) type and infiltrated‐inflamed (I‐I) type. Type I‐E has no CTLs cells in the tumor core. Also known as "cold tumors". There are only a small number of immune cells or inhibitory subsets in their TME, such as Treg, MDSC (Myeloid‐derived suppressor cells), and TAM (Tumor‐associated macrophages), and effector immune cells can not effectively infiltrate into the tumor microenvironment, only distributed in the peripheral matrix, so it is difficult to exert tumor suppressor function. Type I‐I TME is enriched with activated T and myeloid cells and can express chemokines, Type Ⅰ IFN (Interfeon) signals. Immunology is also known as "hot tumor". Characterized by high infiltration of CTLs expressing PD‐1 and expression of immunosuppressive PD‐1 ligand (PD‐L1) of leukocytes and tumor cells. A subclass of I‐I TMEs, termed TLS‐TMEs, displays histological evidence of TLSs (Tertiary Lymphoid Structures), lymphoid aggregates whose cellular composition is similar to that in lymph nodes. It is usually associated with a good prognosis with ICB treatment.^28^, ^29^, ^30^, ^31^ TMEs lead to tumor PD‐1/PD‐L1 monoclonal antibody resistance, which is mainly caused by pathological changes in various elements in the microenvironment. Such as loss of TILs lack of PD‐L1 expression, CD8^+^T cell failure, tumor‐related immunosuppressive cells, and transcriptomic changes in tumor tissues. These immunomodulatory factors affect tumor‐specific immune responses. The following will be elaborated one by one.

### 3.1 Tumor infiltrating lymphocytes (TIL)

TILs is a heterogeneous population in which frequency, location, and subgroup ratio in solid tumors are associated with prognosis and response to immunotherapy.^32^, ^33^ This group of cells contains NK cells, B cells, etc, but most of them are CD3^+^T cells, which are also effector cells of immune response. In TIL cells from different tumor sources, the proportion of CD4^+^T cells and CD8^+^T cells is different, mainly CD8^+^T cells in most cases, which can specifically recognize and kill tumors, and their number and activity determine the effect of antitumor immunity, and also affect the efficacy of ICB. Some studies believe that TIL status is a better predictor of tumor prognosis than histological grade, DNA MMR (Mismatch repair) and BRAF mutations.^34^ The decrease in intratumoral CD8^+^TIL density was significantly correlated with the deterioration of RFS (Recurrence Free Survival).^35^ The presence of CD8^+^ T cells in tumors is a prerequisite for tumor reduction, and chemokine expression plays a key role in the migration of T cells from the circulatory system to the tumor, as found in anti‐PD‐1/PD‐L1 monoclonal antibody therapy for metastatic melanoma.^36^ Epigenetic silencing was used to inhibit the expression of CXCL（CXC Motif Chemokine Ligand）9 and CXCL10 genes of Th_1_ chemokines, which could inhibit the migration of T cells, thus, reducing the infiltration of CD8^+^ T cells in the tumor microenvironment and weakening the effect of immunotherapy. Epigenetic modulators could eliminate such inhibitory effect. Moreover, abnormal tumor signaling pathway is closely related to the decrease in tumor‐specific T‐cell infiltration, which reduces the density of tumor TIL, inhibits the immune response of tumor, and produces PD‐1/PD‐L1 monoclonal antibody resistance.^37^ The specific content is elaborated in the fourth section. In addition, studies have found that NK cell infiltration and STAT1 phosphorylation are typical features of effective microenvironmental immunotherapy in tumor tissues. Using STAT1 activation of cytokines IFNγ(interferon‐γ), TLR3 Ligand Poly (I:C), and anti‐IL‐10 antibodies, the researchers pretreated tumor‐modeled mice, and sensitized the tumors to ICB by attracting the NK cells that produce interferon‐γ into the tumors, thereby increasing the cure rate. These data suggest a biomarker‐driven approach to patient management to predict whether patients will benefit from sensitive treatment with PD‐1/PD‐L1 monoclonal antibody or ICB.^38^, ^39^ Thus, TIL heterogeneity in different tumor tissues hinders the stability of ICB efficacy. Different biological characteristics of immune cell populations suggest that immunotherapy can not be fixed, and the single use of PD1/PD‐L1 blockers is bound to develop extensive drug resistance. According to the individual characteristics of patients, combining tumor chemokines, cytokines, molecular targeting, and other approaches to change the nature of TIL in tumor microenvironment and increase the effective contact between effector cells and tumor cells should significantly promote the clinical efficacy of ICB and improve the prognosis.

### 3.2 Immunosuppressive cells

In the tumor microenvironment, tumor cells interact with immunosuppressive cells, such as Treg, Th2 (T helper 2 cell), MDSC, and TAM, to regulate the occurrence and development of tumors.

Tregs are the main immunosuppressive subsets of CD4^+^T cells, and are a class of T‐cell subsets that can control autoimmune reactivity in vivo. Tumor progression is promoted by the release of immunosuppressive molecules such as TGF‐β (Transforming growth factor‐β), IL‐35, IL‐10, and depleted IL‐2, which inhibit or downregulate the induction and proliferation of effector T cells.^40^, ^41^, ^42^, ^43^, ^44^, ^45^ The tumor can induce treg cells,^46^ promoting CD8^+^T cell failure, removing Tregs from the tumor microenvironment can enhance the antitumor immune effect.^47^ The decrease in CD8^+^T/Tregs ratio can be used as a negative predictor of anti‐PD‐1 monoclonal antibody efficacy.^48^ These data suggest that after immunotherapy, if the tumor does not experience an increase in Teff (T effector cells) and a decrease in Tregs, or if the number of Treg cells in the tumor matrix increases, such patients may be resistant to PD‐1/PD‐L1 monoclonal antibody.

MDSCs (Myeloid‐derived suppressor cells) are a group of heterogeneous cells that inhibit effector T‐cell responses and induce Tregs.^49^ The presence of tumor microenvironment can reduce the effect of immunotherapy.^50^ MDSCs are induced by foreign substances (such as tumor source factors) in immature myeloid cells during their differentiation, which interfere with the production, proliferation, migration, and activation of MDSC. MDSCs can promote the invasion and metastasis of angiogenic tumors, and play an immunosuppressive role mainly through the following factors: IDO (Indoleamine 2,3‐dioxygenase), ARG1 (Arginase‐1), ROS (Reactive oxygen species), IL‐10, iNOS (Inducible Nitric Oxide Synthase), COX‐2 (Cyclooxygenase‐2), NO (Nitric oxide), etc^51^; at the same time, MDSCs can also recruit Tregs to the tumor microenvironment to jointly play the role of immunosuppression. In addition, studies have shown that inhibition of PI3K also has a synergistic effect with immunocheckpoint inhibitors. In mice with failed PD‐1 monoclonal antibody treatment, inhibition of PI3K can reduce the circulation and recruitment of MDSCs, inhibit immunosuppressive factors such as IL‐10 and TGF‐β, promote the production of inflammatory mediators IL‐12 and INF‐γ, and achieve the same effect of combined inhibition of CTLA‐4 and PD‐1 monoclonal antibodies.^52^, ^53^ These studies suggest that PI3K inhibitors can be used as a potential therapeutic target in combination with PD‐1/PD‐L1 antibody to combat single‐use drug resistance. In terms of metabolism, arginine metabolism provides energy for MDSCs with the involvement of ARG1, and the loss of ARG1 activity can downregulate the inhibition efficiency of MDSCs and improve the sensitivity of PD‐1/PD‐L1 antibody.^54^

TAMs are another group of cells that influence the effectiveness of immunotherapy. TAMs include M_1_‐like macrophages involved in promoting anti‐tumor immunity and M_2_‐like macrophages with cancer‐promoting properties. PD‐1 can be expressed on the membrane of TAM, M_2_‐like macrophages express more PD‐1 than M_1_‐like macrophages, and PD‐1^‐^TAM is mainly shown as M_1_ phenotype.^55^, ^56^ The number of PD‐1^+^M_2_‐like macrophages increased with disease stage, suggesting that PD‐1^+^M_2_‐like macrophages may accumulate in the tumor microenvironment over time.^57^ M_2_‐like macrophages can mediate the immune escape of tumor cells through PD‐1, and can be activated by IL‐4, IL‐10, IL‐13, or CSF1 (Colony stimulating factor 1), and participate in wound healing and tissue repair, and mediate the anti‐inflammatory response by producing anti‐inflammatory cytokines including IL‐10.^58^ Tumor invasion and metastasis were promoted by angiogenesis and extracellular matrix remodeling.^59^ Clinical studies have found that high levels of TAMs are associated with poor prognosis in human cancers.^60^ In the mouse model of lung adenocarcinoma, the inactivation of CCL2 (C‐C Motif Chemokine Ligand 2) and CCR2 (C‐C Motif Chemokine Receptor 2) signals can reduce the recruitment of M_2_ macrophages and inhibit tumor growth.^61^ To overcome the potential resistance associated with macrophages, blocking macrophage CSF‐1R (Colonies stimulates factor 1 receptor), reduces the frequency of TAMs, increases IFN production, and increases tumor cell response to drugs in a mouse model of pancreatic cancer. More importantly, CSF‐1R blockers combined with PD‐1 or CTLA‐4 antibodies, combined with gemcitabine, were more effective.^62^

To sum up, the inhibitory immune cells in tumors also show heterogeneity and are affected by different factors in the tumor microenvironment, such as chemokines, cytokines, and colony‐stimulating factors. The proportions of M_1_ and M_2_ TAM in tumors are more conducive to tumor proliferation due to the influence of TME. This heterogeneous mechanism limits the scope of clinical use of PD‐1/PD‐L1 blockers alone. From the perspective of inhibitory immune cells promoting ICB resistance alone, a series of indicators in TME may predict drug resistance mechanism, such as CD8^+^/Tregs ratio, IDO, ARG1, CSF‐1R, M_1_/M_2_ ratio, etc For these indicators, combined therapy may achieve better clinical treatment effect and prognosis. The combined use of drugs against immunosuppressive cells includes IDO inhibitor, ARG1 inhibitor, PI3K inhibitor, and ICB. Clinical trials observed that the combination of the two inhibitors had a positive effect on the improvement of treatment indicators, with controllable adverse reactions and good clinical compliance. However, there are few reports on three or more combined applications.

### 3.3 Immune factors

Immunosuppressive factors in TME are mainly released by tumors or macrophages for local inhibition of antitumor immunity. Among them TGF is particularly important. TGF‐β can stimulate Tregs to produce immunosuppressive effects, and its elevation is associated with poor prognosis in a variety of tumors.^63^ However, single drug inhibition of TGF‐β signal has limited efficacy in clinical trials and failed to effectively promote antitumor immune response, which is because inhibition of TGF‐β signal can promote tumor PD‐L1, PD‐L2 expression upregulation, and MDSCs recruitment, promoting antitumor immune resistance; treatment with PD‐1 antibody alone can improve the CD4^+^Treg/CD4^+^T ratio; and increase the expression of pSmad (Phospho drosophila mothers against decapentaplegic)3 in tumor cells, while the addition of TGF‐β inhibitor antibody can eliminate these adverse factors.^64^ Studies have shown that inhibition of both TGF‐β and PD‐L1 receptors can reduce the tumor phenotype and improve survival and inhibit tumor development.^65^

Furthermore, specific chemokines and chemokine receptors transport MDSCs and Tregs to the tumor microenvironment. For example, the tumor secretes CCL2, CCL5, CCL7, and CXCL8, which, after binding with the receptors CCR1 or CXCR (Chemokine C‐X‐C‐Motif Receptor)2 on MDSCs, Treg, and M_2_‐type macrophages, attract immunosuppressive cell aggregation in the tumor microenvironment, and mediate the occurrence of resistance to PD‐1/PD‐L1 blockers by inhibiting Teff function.^66^ Thus, inhibitors of these chemokine receptors can prevent the immune escape of tumor cells and improve the antitumor response of T cells.^67^ CXCR4 is an evolutionary highly conserved GPCR (G‐protein coupled receptor) expressed in peripheral mononuclear, B cells, and naive T cells. CXCR4 is overexpressed in more than 23 types of human cancer and controls its metastasis in most of the overexpressed tumors.^68^ Blocking the CXCR4‐CXCL12 axis as a therapeutic target has a benign effect on TME, which can reverse the immunosuppressive cells rich in TME. Such as Treg and M_2_ and N_2_ (Neutrophils Type 2) polarization of immune tolerance.^69^, ^70^, ^71^ It was found that CXCR4 antagonist could regulate the effect/regulate cell pathway and function in TME, enhance the anti‐PD‐1 effect by manipulating the transport of immune cells, inhibit the inherent PD‐1 function of human melanoma xenograft tumor cells, and enhance the independent response of T cells, providing support for the combination of PD‐1/PD‐L1 immunotherapy.^72^ Other studies have suggested that the CXCR3 chemokine system in tumor does not chemotactic peripheral CD8^+^T cells to tumor invasion, but enhances the functional activity of CD8^+^T cells in tumor. For PD‐1 monoclonal antibody to work, CXCL9 from CD103^+^ dendritic cells and CXCR3 from CD8^+^T cells are required; CXCR3 ligands (CXCL9 and CXCL10) are positive indicators of anti‐PD‐1 response; Induction of CXCR3 ligand in nonreactive tumors restores the sensitivity to anti‐PD‐1. Inhibition of any component of the CXCR3 chemokine system may impair the therapeutic efficacy of PD‐1. The removal of CXCL9 and CXCL10 gene inhibition by epigenetic modulators can transform the anti‐PD‐1 nonreactive tumor into reactive tumor, which is related to the induction of these chemokines by the DC in the tumor. These data suggest that tumor‐specific induction of these potent chemokines in nonreactive tumors may be a viable therapeutic strategy to amplify the benefits of anti‐PD‐1 therapy.^73^ But other studies suggest that the CXCR3 chemokine system in tumor does not chemotactic peripheral CD8^+^T cells to tumor invasion, but enhances the functional activity of CD8^+^T cells in tumor. This conclusion does not affect the synergistic effect of CXCR3 system on PD1/PD‐L1 antibody.

EV (Extracellular vesicles) in tumor tissues also have immunocytosuppressive effects. One small EV, containing ARG1 was found in tumors, ascites, and plasma in patients with OvCa (ovarian cancer), this EV reduces the expression levels of CD3 ζ and CD3 ε chains on the membrane and inhibits CD4^+^ and CD8^+^T cell proliferation in vivo and in vitro. And the EV can transport its ARG1 to other sites in the body, such as draining lymph nodes, thus, promoting immunosuppression and accelerating tumor progression. It was found that ARG1^+^EV could be endocytosis by DC cells, thus, inhibiting the stimulating effect of DC cells on T cells, and the use of ARG1 inhibitor could reverse the inhibitory effect.^74^ Moreover, it was found that the expression of exosome PD‐L1 in plasma was significantly correlated with the treatment response of ICB and could be dynamically measured, which might provide useful information for the treatment response of PD‐1 antibody.^75^

Some studies also found that CD38 expression was significantly upregulated when PD‐1/PD‐L1 antibody was used to treat KRAS/P53 mutant lung tumors, which promoted therapeutic resistance. Genetic and proteomic analysis showed that the reactivated immune response after blocking treatment with PD‐1/PD‐L1 antibody led to upregulation of CD38.^76^ CD38 is a member of the ribocyclic enzyme family, a well‐characterized extracellular enzyme with a variety of functions, both enzyme protein and cell surface expression of the receptor.^77^ It is an important catalytic enzyme in the adenosine production process, which can inhibit the proliferation and secretion of CD8^+^T cells and the tumor killing effect through adenosine receptor 2A or 2B signaling cascade pathway.^78^ In tumor cells resistant to PD‐1/PD‐L1 blockers, CD38 mRNA and protein levels were significantly increased, and the combination of anti‐CD38 and anti‐PD‐1/PD‐L1 treatment could improve the antitumor immune response, revealing the main mechanism of CD38 related to acquired resistance of PD‐1/PD‐L1 blockers.^76^ In recent years, the physiological and pathological effects of external vesicles and their metabolites in the humoral circulation have been paid more and more attention by researchers. Secretory of EV in tumor microenvironment plays an important role in maintaining the survival of tumor cells. In most cases, ICB may not activate CD8^+^T cells. Under the influence of metabolites and external vesicles secreted by tumor cells, Teff function fails and ICB resistance is generated. Therefore, an in‐depth study on the external vesicles equipped with immune influences was conducted to selectively and comprehensively treat the active proteins or other substances in the external vesicles, such as inhibition of ARG1 and CD38, to activate the body or local immune function of patients, providing a new treatment idea.

### 3.4 Severe depletion of CD8^+^T cells

Sustained antigen stimulation in the generation of drug‐resistant tumors or chronic inflammation can cause T cells to form a failure phenotype, which is manifested by decreased cytokine secretion of immune dysfunction and continuous expression of surface inhibitory receptors, known as depleted T cells.^79^ Continuous stimulation of antigen is the main reason for the generation of depleted T cells, and the expression of PD‐1 plays an important role in the maintenance of the dysfunctional state of depleted T cells, its persistent upregulation indicated that the immune function was continuously impaired. Different from functional effector or memory T cells, depleted T cells have decreased proliferation ability and cytotoxic activity, and subsequently abnormal or even absent cytokines.^80^ In the early stage, IL‐2 production was absent, the ability of killing target cells was decreased, and the ability of cloning proliferation was impaired, but TNF (Tumor Necrosis Factor)‐α was still produced. In the late stage of depletion, the production capacity of IFN‐γ was obviously impaired, and the immune function was seriously impaired. Failing CD8^+^T cells with moderate expression of PD‐1 can be reversed by anti‐PD‐1/PD‐L1 monoclonal antibody, but not by cells with high expression of PD‐1.^81^, ^82^ The ratio of failing CD8^+^T cells with moderate expression of PD‐1 to severely failing CD8^+^T cells with high expression of PD‐1 may be a key indicator to reverse resistance of PD‐1/PD‐L1 antibody. The failing T cells treated with PD‐1 monoclonal antibody have something in common with effector T cells, and they have short‐term functional gains. However, they have little in common with memory T cells, and they cannot become memory T cells through antigen clearance, and they will fail again. This phenomenon may be related to the epigenetic stability of failing T cells and is one of the causes of acquired drug resistance. The combination of immunocheckpoint inhibitors and T‐cell epigenomic engineering or epigenetic modulators may provide an opportunity for lasting effectiveness of immunotherapy.^83^

Depletion of CD8^+^T cells is the most important part of tumor immune tolerance. Persistent tumor antigens that cannot be completely eliminated for a variety of complex reasons may eventually cause persistent changes in the epigenetic stability of effector cells, making them less sensitive to tumor antigens presented and inducing "immune desensitization". With the extension of tumor existence time in vivo, the faster tumor proliferation, the more tumor antigen proteins, the higher the expression of PD‐L1, the more failure of effector T cells, the worse the immune sensitivity, and the greater the impact on the efficacy of PD‐1 antibody. Combined with epigenetic regulators, ICB resistance may be removed.

### 3.5 Tumor transcriptome and epigenetic changes

Biopsy specimens of melanoma patients before treatment with PD‐1/PD‐L1 monoclonal antibody were collected for classification and comparison according to the effect at the later stage of treatment. It was found that some genes were highly expressed in patients with failed treatment. These include epithelial‐mesenchymal transition‐related genes (*AXL, ROR2, WNT5A, LOXL2, TWIST2, TAGLN, FAP*), monocyte macrophage chemokine genes (*CCL2, CCL7, CCL8, CCL13*), immunosuppressive factor genes (*VEGFA, VEGFC, IL10*), and damage repair, angiogenesis‐related genes^84^ And this feature is widely found in a variety of tumors. Exon and transcriptome sequencing of tumor tissues before treatment is of great value in predicting anti‐PD‐1 treatment response.

Genetic and epigenetic changes lead to the production of immunogenic‐specific antigens on the tumor cell surface. Such changes frequently occur in various tumors, which is the prerequisite for tumor immunotherapy and an important molecular basis for ICB resistance. It has been confirmed that resistance to PD‐1/PD‐L1 monoclonal antibody is associated with tumor immune escape, mostly due to epigenetic changes in tumor cells. For example, malignant cells selectively reduce or silence the expression of TAA (Tumor‐associated antigen), HLA (Human leukocyte common antigen), and costimulatory molecules, so as to evade recognition by host immune system^85^ resulting in resistance to immunocheckpoint blockers. Epigenetic changes in tumors are commonly seen in the methylation of CpG island in the promoter region of tumor suppressor genes. Histone acetylation, methylation, phosphorylation, ubiquitination, ADP ribosylation, and non‐coding RNA can all affect the transcriptional activity of genes. Epigenetic groups have become new targets for individualized treatment of tumors.^86^ The establishment of tumor immune resistance in epigenetics is also related to miRNA, and they can be classified as promoters or antagonists of drug resistance according to their different modes of action. Different members of the miR‐8 family (miR‐200a, b, and c) can also target the inhibition of PD‐L1 gene expression in lung cancer, leading to increased CD8^+^T cell activation and tumor immune monitoring. The researchers also observed that EMT (Epithelial‐Mesenchymal Transition) activator ZEB1 (Zinc Finger E‐Box Binding Homeobox 1) mediates the transcriptional inhibition of miR‐200, thereby removing the inhibition of miRNA on the expression of PD‐L1 in tumor cells, leading to CD8^+^T cell failure.^87^ Therefore, the downregulation of miR‐8 family may be related to the resistance of PD‐1/PD‐L1 monoclonal antibody. There is increasing evidence that abnormal epigenetic modifications, silencing effector T‐cell chemokines, play an important role in cancer expression.^88^, ^89^ Histamine LSD1 (Lysine‐specific demethylase 1) inhibits the reexpression of chemokines such as CCL5, CXCL9, and CXCL10 by increasing the level of H3K4me2 in the proximal promoter region, thereby preventing CD8^+^T lymphocytes from migrating to the tumor microenvironment. In mice with TNBC (Triple‐negative breast cancer cells) xenograft tumor, the treatment effect of anti‐PD‐1 antibody alone was not obvious, while LSD1 inhibitor combined with PD‐1 antibody could significantly inhibit tumor growth and lung metastasis.^90^ Epigenetic properties that can be reversed under certain conditions also provide new opportunities for tumor treatment. HDACi (Histone deacetylase inhibitors) are a new class of anticancer drugs that induce transient changes in gene expression on a large scale, without involving permanent changes in DNA sequences.^91^, ^92^ HDACi time‐dependent upregulation of PD‐L1 mRNA and protein expression in TNBC. In vitro coculture of HDACi and PBMCs (Peripheral blood mononuclear cells) can upregulate PD‐L1 and HLA‐DR of tumor cells and downregulate CD4^+^Foxp3^+^Treg. In animals, HDACi significantly enhanced the anti‐PD‐1/CTLA‐4 response in mice with triple‐negative breast cancer. Therefore, in the tumor microenvironment, increased tumor infiltration of T cells, improvement of PD‐1/PD‐L1 axis effect, and reduction in CD4^+^ Foxp3^+^ T cells, weaken the mechanism of ICB resistance, suggesting that HDACi combined with immunocheckpoint inhibitor is a promising therapeutic strategy.^93^ The EZH2 (Enhancer of Zeste 2 Polycomb Repressive Complex 2 Subunit) catalytic subunit of PRC2 (Polycomb repressive complex 2) is a highly conserved histone methyltransferase. It mediates trimethylation of lysine 27 on histone 3 (H3K27me3) to induce chromatin compaction and transcriptional inhibition of target genes.^94^, ^95^ Several studies have shown that EZH2 plays an important role in the development and progression of cancer. In many cancers, EZH2 overexpression may be due to a functional gain mutation in tyrosine 641 or a functional loss mutation in an EZH2 antagonist.^96^, ^97^ In uveal melanoma, EZH2 overexpression is associated with an ineffective T‐cell response.^98^ Specific interference with the *EZH2* gene in Treg‐induced tumor response to immunotherapy.^99^ After treatment with EZH2 inhibitor, the effector cytokines of Th_1_ type, CD8+ T, and other cells were recovered, and the tumor response to immune blockers was enhanced.^100^ With the increase in DNMTs (DNA methyltransferases), the tumor suppressor protein *PDLIM2* gene promoter is blocked from expression due to hypermethylation in lung cancer, and the treatment with DNMT inhibitor 5‐AZA‐DC can lead to the promoter hypommethylation and the reexpression of PDLIM2 in human lung cancer cells.^101^ Epigenetic modifiers also include BETi, IDHi, DOTi, etc, and clinical treatment studies in combination with PD‐1/PD‐L1 are in full development. See Table 1.^102^

**TABLE 1:** Clinical trials of epigenetic modification agents combined with immunotherapy

Clinical Trials Identifier	Recruitment Status	Phase	Cancer Type	Immunotherapy agent	Epigenetic drug(s)	Other agents
NG03993626	Active, not recruiting	I/II	Colorectal (microsatellite stable)	Nivolumab (PD‐1)	CXD101 (HDACi)	
NG03978624	Recruiting	II	Bladder Cancer	Pembrolizumab (PD‐1)	Entinostat (HDACi)	
NCT03903458	Recruiting	I	Melanoma	Nivolumab (PD‐1)	Tinostamustinc (HDACi fusion protein)	
NCT03854474	Recruiting	I/II	Urothelial, bladder	Pembrolizumab (PD‐1)	Tazemetostat (competitive inhibitor EZH2)	
NCT038127%	Recruiting	II	GI cancer	Avelumab (PD‐L1)	Domatinostat (HDAC inhibitor)	
NCT03765229	Recruiting	II	Melanoma	Pembrolizumab (PD‐1)	Entinostat (HDACi)	
NCT03612739	Recruiting	I	AML, myelodysplastic syndromes	Chimeric Antigen Receptor T Cells	Azacitidine (DNMTi)	
NCT03280563	Recruiting	I/II	Breast cancer	Atezolizumab (PD‐L1)	Entinostat (HDACi)	
NCT03250273	Recruiting	II	Cholangiocarcinoma, Pancreatic Adenocarcinomas	Nivolumab (PD‐1)	Entinostat (HDACi)	
NCT03240211	Recruiting	I	Peripheral T‐Cell Lymphoma, Cutaneous T‐Cell Lymphoma	Pembrolizumab (PD‐1)	Decitabine (DNMTi)	
NG03220477	Recruiting	I	Lung Cancer	Pembrolizumab (PD‐1)	Guadecitabine (DNMTi) Mocetinostat (HDACi)	
NCT03179943	Recruiting	II	Urothelial cancer	Atezolizumab (PD‐L1)	Guadecitabine (DNMTi)	
NCT03150329	Recruiting	I	Lymphoma	Pembrolizumab (PD‐1)	Vorinostat (HDACi)	
NCT02959437	Active, not recruiting	I/II	Solid Tumors, Metastatic cancers	Pembrolizumab (PD‐1)	Epacadostat (HDACi)	
NCT02951156	Active, not recruiting	Ⅲ	Diffuse Large B‐Cell Lymphoma	Avelumab (PD‐L1)	Azacitidine (DNMTi)	
NCT02936752	Recruiting	I	Myelodysplastic syndrome	Pembrolizumab (PD‐1)	Entinostat (HDACi)	
NG'02 900 560	Active, not recruiting	II	Epithelial Ovarian Cancer	Pembrolizumab (PD‐1)	CC‐486 Oral Azacitidine (DNMTi)	
NCT02805660	Active, not recruiting	I/II	Advanced cancer	Durvalumab (PD‐L1)	Mocetinostat (HDACi)	
NC1.03257761	Recruiting	I	Hepatobiliary cancers	Durvalumab (PD‐L1)	Guadecitabine (DNMTi)	
NCT02697630	Active, not recruiting	II	Metastatic Uveal Cancer	Pembrolizumab (PD‐1)	Entinostat (HDACi)	
NCT026195253	Recruiting	I	Renal cell carcinoma	Pembrolizumab (PD‐1)	Vorotinostat (HDACi)	
NCT02497404	Active, not recruiting	II	Leukemia, myelodysplastic syndromes	Alemtuzmab (CD52)	Azacitidine (DNMTi)	TBI, fludarabine, melphalan
NCT02518958	Active, not recruiting	I	Lymphoma, malignant solid tumors	Nivolumab (PD‐1)	RRx‐001 (DMNTi)	
NCT02845297	Recruiting	II	AML	Pembrolizumab (PD‐1)	Azacitidine (DNMTi)	
NCT03525795	Active, not recruiting	I/II	Solid tumors	Ipilimumab (CTLA‐4)	CPI‐1205 (EZH2i)	
NCT02512172	Active, not recruiting	I	Colorectal cancer	Pembrolizumab (PD‐1)	Romidepsin	
NCT02453620	Recruiting	I	Breast cancer	Nivolumab (PD‐1), Ipilimumab (CTLA‐4)	Entinostat (HDACi)	
NCT02419417	Recruiting	I/II	Solid Tumors, Hematologic malignancies	Nivolumab (PD‐1)	BMS‐986158 (BETi)	
NCT02395627	Active, not recruiting	II	Breast cancer	Pembrolizumab (PD‐1)	Vorotinostat (HDACi)	Tamoxifen
NCT02250326	Active, not recruiting	II	NSCLC	Duravalumab (PD‐1)	CC‐486 Oral Azacitidine (DNMTi)	Nab‐paclitaxel
NCT02%1101	Recruiting	I/II	Solid Tumors, Metastatic cancers	Anti‐PD‐1 Antibody	Decitabine (DNMTi)	Chemotherapy
NCT01928576	Recruiting	II	NSCLC	Nivolumab (PD‐1)	Azacitidine, Entinostat (HDACi)	
NCT03019003	Recruiting	I/II	Head and neck cancer	Tremelimumab (CTLA‐4) Durvalumab (PD‐L1)	Azacitidine (DNMTi)	
NCT02546986	Completed	II	Non–small cell carcinoma	Pembrolizumab (PD‐1)	CC‐486 Oral Azacitidine (DNMTi)	
NCT02608437	Completed	I	Melanoma	Ipilimumab (CTLA‐4)	Guadecitabine (DNMTi)	
NCT01038778	Completed	I/II	Renal cell carcinoma	IL‐2	Entinostat (HDACi)	

Note

List of active and accruing clinical trials combining epigenetic and immune therapy. Overview of ongoing and completed clinical trials of epigenetic drugs in combination with immunotherapy.

It can be seen from the above that transcriptional and epigenetic changes can regulate the expression of various proteins in the tumor microenvironment and play a decisive role in drug resistance of PD‐1/PD‐L1 blockers. The inhibition of tumor suppressor genes and the overexpression of proto‐oncogenes in tumor cells are conducive to the proliferation, metastasis, and drug resistance of tumor cells. In TIL, immune effector cells and inhibitory cells and their cytokines are affected by epigenetic changes, forming an immune escape microenvironment more conducive to tumor growth; in collaboration with other immune injury mechanisms (such as mutation‐induced protein dysfunction, including inhibitory immune checkpoint proteins, costimulation signaling pathway proteins, and proteins associated with antigen presentation pathway chains, etc) to further promote tumor progression. Therefore, the single use of PD‐1/PD‐L1 blocker in clinical tumor therapy is no longer sufficient. Detection techniques of genome, transcriptome, methylation group, and acetylation group provide powerful diagnostic conditions for understanding tumor gene or epigenetic changes. Reasonable combination of ICB and epigenetic modifier DNMTi, HDACi, EZH2i, and other targeted drugs with various proven tumor drug resistance mechanisms may achieve unexpected short‐term or long‐term efficacy on the premise of careful observation of adverse reactions and guarantee of patients' treatment compliance.

### 3.6 Tumor PD‐L1 expression level and anti‐PD‐1/PD‐L1 resistance

In most tumor tissues, patients with positive PD‐L1 expression have better clinical response to PD‐1/PD‐L1 monoclonal antibody therapy.^103^, ^104^, ^105^, ^106^ After receiving PD‐1 antibody treatment in melanoma patients, tumor biopsy showed that the early expression of PD‐L1 on tumor cells could improve the efficacy of PD‐1 antibody.^107^ The mechanism affecting PD‐L1 expression involves induction of JAK/STAT signal, loss of PTEN (Phosphate and tension homology deleted on chromsome ten), PI3K and/or AKT mutations, EGFR (Epidermal growth factor receptor) mutations, *MYC* (Myelocytomatosis oncogene) overexpression, frequent amplification of chromosome 9p24.1 region, and increase in PD‐L1 transcription level, etc.^108^, ^109^, ^110^, ^111^, ^112^, ^113^ Transcription factors AP‐1 (Dimer transcription factor complex activator protein‐1) and YY1 (The ubiquitous transcription factor Yin Yang 1) also had significant effects on the expression of PD‐L1. AP‐1 is a family of four subfamilies of transcription factors: Jun (C‐Jun, JunB, JunD), Fos (C‐Fos, FosB, Fra1, Fra2), Maf (Myofascial fibrosarcoma) (C‐Maf, MafB, MafA, Mafg/f/k, Nrl), and ATF‐activated transcription factor (ATF2, LRF1/ATF3, BATF, JDP1, JDP2).^114^ AP‐1 is a group of proteins widely involved in cellular processes and is a key regulator of nuclear gene expression during T‐cell activation. It is also one of the downstream targets of MAPK (Mitogen‐activated protein kinase) signal cascade. In melanoma cells resistant to BRAF inhibitors, C‐Jun activity was increased, and the activation of MAPK promoted the expression of PD‐L1. Inhibition of C‐Jun expression by siRNA resulted in decreased or almost complete inhibition of PD‐L1 expression in many drug‐resistant cell lines.^115^ AP‐1 also binds with other transcription factors, such as NFAT (Nuclear factor of activated T cells), to regulate a variety of immune‐promoting cytokine genes.^116^ YY1 is a zinc finger transcription factor belonging to the Polycomb Group protein family, and one of the mechanisms by which it regulates tumor resistance to cytotoxic immune function is by regulating the expression of PD‐L1 on tumor cells. YY1 may enhance the expression of PD‐L1 by downregulating the activity of P53. YY1 downregulates P53 by inhibiting the interaction between P53 and p300 and enhancing the interaction with Mdm2 (Murine double minute 2). When P53 is inhibited, it can no longer induce miR‐34a transcription, and miR‐34a can no longer degrade PD‐L1. Moreover, YY1 can also be through the cytokines IL‐6, IL‐17, TGF‐β, and IFN, the signaling pathway PTEN/PI3K/AKT/mTOR (Mammalian target of rapamycin), c‐Myc, COX‐2, etc, regulates PD‐L1.^117^ However, not all patients with positive PD‐L1 expression responded to PD‐1/PD‐L1 monoclonal antibody. This may be due to the lack of proper Teff infiltration in the tumor microenvironment and the inability of the PD‐1/PD‐L1 pathway to be established. Because the expression of tumor PD‐L1 is not only induced by the interferon‐γ(IFN‐γ) secreted by Teff cells but also driven by the signal of tumor proto‐oncogene. Thus, PD‐1/PD‐L1 monoclonal antibody is resistant due to the lack of antitumor effector cells.^118^, ^119^ Or it may be that overexpression of PD‐L1 causes severe failure of CD8^+^T in local tissues, leading to drug resistance. The establishment of a comprehensive model based on the expression of tumor PD‐L1 and the characteristics of immune cell infiltration can better predict which patients can benefit from anti‐PD‐1/PD‐L1 monoclonal antibody therapy. The tumor microenvironment was classified into four types according to PD‐L1 expression and TIL infiltration abundance: type Ⅰ, acquired immune resistance type (PD‐L1^+^, TIL^+^), and the best anti‐PD‐1 treatment response. Type Ⅱ, immune neglect type (immune desert type) (TIL^‐^, PD‐L1^‐^), the worst anti‐PD‐1 response; TypeⅢ, intrinsic induction type (TIL^‐^, PD‐L1^+^), recommended to combine with other treatment methods to promote the increase in TIL in the microenvironment and improve the anti‐PD‐1 treatment response. TypeⅣ, how Type (TIL^+^, PD‐L1^‐^), the need to remove other factors that inhibit the immune response in microenvironment.^120^

There are many reasons that can induce the expression of PD‐L1, which can be induced at different molecular levels. From signaling pathway activation, protein abnormalities, to gene mutations, transcription factors, and miRNA regulation, etc, it involves multiple levels of protein, epigenetic, and gene changes. In the tumor microenvironment, PD‐L1^‐^ produces resistance to PD‐1/PD‐L1 blockers. Theoretically, PD‐1/PD‐L1 block therapy is of low therapeutic value for such tumors. However, tumor heterogeneity determines that PD‐L1^‐^ is not absolute in tumor tissues, especially in the process of pathological changes or under the pressure of drug therapy, PD‐L1 still has the possibility of turning positive. Dynamic observation of its changes is conducive to the in‐depth study of the tumor. Meanwhile, in terms of treatment, it is still a reasonable choice to combine PD‐1/PD‐L1 antibody therapy in stages.

### 3.7 Effects of intestinal flora on resistance to PD‐1/PD‐L1 monoclonal antibody

The microbiome helps to form an integrated immune system.^121^ The effects and possible mechanisms of combined therapy as a synergistic ICBs are still being investigated. Studies have found that intestinal microbiota disorders can lead to reduced efficacy of ICBs (increased drug resistance).^122^ Analysis of the microbiome showed that bifidobacteria were dominant in mice with delayed tumor growth and a favorable response to PD‐1‐based treatment. Oral probiotics supplemented with bifidobacterium can restore the antitumor efficacy of PD‐L1 blockers in mice with “adverse” intestinal microbiota, which may be caused by increased tumor‐specific CD8^+^T cell activity by enhancing DC maturation.^123^ Other studies have also demonstrated the role of gut microbiota in the treatment of ICB.^124^, ^125^, ^126^ Due to the impact of antibiotics on intestinal flora, NSCLC (Non‐small cell lung cancer), patients who received antibiotics in the first 2 months or 1 month after their first ICB treatment had significantly lower PFS and OS (Overall survival) compared with patients who were not treated with antibiotics.^122^ The antibacterial action of antibiotics can inhibit the clinical efficacy of ICBs in patients with advanced cancer. Transplanting fecal microorganisms from ICBs sensitive cancer patients into sterile mice or antibiotic‐treated mice can improve the antitumor effect of PD‐1 blockers. The results showed that the clinical efficacy of ICBs was related to the relative abundance of Akkermansia muciniphila. After transplantation of fecal microorganisms from patients without clinical efficacy, oral A. muciniphila supplement can increase the recruitment of CCR9^+^CXCR3^+^CD4^+^T cells in tumor tissues of mice in DC and IL‐12‐dependent manner, and restore the efficacy of PD‐1 blocker.^122^ Other studies suggest that intestinal flora can affect T‐cell immune response and alter TME in different degrees. The specific mechanisms need to be evaluated from the multidimensional perspective of the tumor, such as TME and host characteristics involved in therapeutic effects.^127^ It may be related to metabolic changes caused by microbiota that regulate TME changes and enable T‐cell function recovery to offset tumor‐induced immune tolerance.^128^ Diet changes, therefore, may be an effective intervention measures, regulating the symbiotic entity by providing more specific essential nutrients to promote the expansion of beneficial bacteria, or by reducing the nutrient supply makes the harmful bacteria is eliminated and the intestinal flora balance, beneficial to reverse TME, restore Teff function, strengthen the PD‐1/PD‐L1 antibody sensitivity, and inhibit tumor growth.^129^

In terms of mechanism, whether promoting DC cell maturation or TME change, the effect of probiotics in intestinal microbiota on tumor immunity has been paid more and more attention. Studies on the correlation between the efficacy of ICBs and intestinal flora provide a new way to solve the problem of drug resistance. Clinical use of combined probiotics has a high expectation for improving the antitumor pharmacological effect of ICBs. There seems to be some correlation between the diversity and stability of intestinal flora and the heterogeneous growth of tumors, and immune surveillance and clearance may be the link between them. The change in intestinal flora abundance has a correlation effect on the prognosis of tumor, and the higher the flora abundance, the better the prognosis. The interaction between intestinal microbiota and immune system not only maintains the tolerance of symbiotic bacteria and food antigens but also enables the immune system to recognize and attack pathogenic bacteria and prevent their invasion. Since the intestinal probiotics do not produce obvious pathological stimulation to the host, the second signal pathway of the immune system costimulation signal is not activated, which will not induce the immune effect. This diversity of antigens provides a wide communication channel between the immune system and in vitro antigens and provides an effective guarantee for the adaptability of the body to the environment and the self‐regulation of immune balance. Heterogeneous tumors also have antigen diversity and their abundance increases with the increase in TMB. As the tumor progresses, pathological changes occur in the lesion site, which can stimulate the immune costimulation signal (the second signal). However, due to various immune escape mechanisms in tumor cells (such as antigen presentation disorder, "immune brake", etc), immune cells cannot be activated, so that tumor cells can avoid immune attack. The high abundance of probiotics in the gut gives the immune system a very high abundance of epitopes. The effector cells are activated by an antigen in the gut that has a similar epitope structure to tumor cells. With the aid of a second signaling system, immune attacks are produced against similarly structured epitopes. At this point, the efficacy of ICBs can be enhanced to combat drug resistance.

### 3.8 Tertiary lymphatic structures (TLS)

TLSs (Tertiary lymphoid structures) are ectopic lymphoid organs that develop in nonleucoid‐like tissues in chronically inflected sites, including tumors. Studies of tumor‐associated lymphocytes have shown that TLS has all the characteristics of normal lymph node formation and produces an antitumor immune response.^130^, ^131^, ^132^ In most cases, the presence of TLS in human solid tumors is critical to the formation of a favorable immune microenvironment to control tumor development. They trigger T cells, activate B cells, and differentiate into plasma cells, making them precise factories for producing antibodies.^133^ The occurrence of TLS is associated with a reduced risk of recurrence of various solid tumors and improved OS.^134^ TLS promoted increased invasion of CD3^+^, CD8^+^, CD20^+^, and decreased invasion of Foxp3^+^ and CD68^+^ cells in the tumor. In TLS^+^ cases, the density of inhibitory immune checkpoint: PD‐1^+^, TIM‐3^+^, and LAG3^+^, was lower. Mature TLS and its degree of maturity are associated with a reduced risk of early HCC recurrence, and are independent prognostic factors for early HCC recurrence. Mature TLS can induce an enhanced antitumor immune response.^135^ Mechanistically, CD8^+^ cytotoxic effector T cells produced in TLSs synergistically interact with B cells to kill tumor cells directly, and can also be activated by macrophage and/or natural killer cell‐mediated ADCC (Antibody‐dependent cytotoxicity) and local complement, making it possible to kill tumor in situ.^136^ B cells have a variety of roles in inhibiting or promoting the immune system's ability to kill tumor cells, depending on whether they are located in immature or mature structures of TLS. B cells in mature TLS are associated with increased T‐cell activity, improving the ability of the immune system to target tumor cells, and increasing the likelihood that tumors will respond to immunotherapy. In patients with metastatic melanoma and renal cell carcinoma, B cells in TLSs have been found to respond to ICB, and the same response characteristics of memory B cells and plasma cells may also contribute to the T‐cell effector response after ICB. Memory B cells may act as antigen presenting cells, driving the expansion of memory and naive tumor‐associated T‐cell responses. B cells also secrete cytokines (including TNF, IL‐2, IL‐6, and IFN‐γ) by activating and recruiting other immune effector cells, including T cells. The transformed memory B cells (which can differentiate into plasma cells) observed in responders suggest that they may potentially contribute to the fight against the tumor response by producing antitumor antibodies. This is an important area of research and further understanding may lead to new therapies to enhance response to ICB.^137^ These results suggest that immunoregulatory therapy targeting TLS may be an effective potential strategy for immune‐mediated tumor suppression. And it provides a new way to study the mechanism of PD‐1/PD‐L1 resistance, especially the potential regulatory effect of antigen presentation and cytokines secreted by B cells on T‐cell effects after ICB resistance. The number and maturity of TLS in tumors may also be a good universal biomarker for the prognosis of various tumors, which also opens a new path for its new target therapy.

The existence of TLS provides a new outpost for tumor immunotherapy, which can closely monitor the development and changes in tumors, cultivate and provide a large number of front‐line effector cells, antigen presenting cells, and tumor antibodies to attack and destroy tumor tissues, and is associated with good prognosis. Immunocheckpoint therapy can maintain TLS regeneration and transform immunocompromised tumors into immunogenic tumors. In addition, other ICB‐insensitive tumors, such as immunodesert, chronic inflammation, or immunosuppressive tumors, can be combined with chemoradiotherapy, molecular‐targeted therapy, oncolytic virus or anti‐angiogenesis, anti‐inflammatory, and immunostimulative therapy. Induce chemokines, cytokines, and engineering DCs that mediate newborn TLS, and finally reconstruct more newborn TLS. It can further enhance the function of PD‐1/PD‐L1 blocker, stimulate immune clearance, reduce the occurrence of drug resistance, and form a virtuous cycle in treatment. Therefore, the analysis of TLS, ICB drug resistance, and antitumor mechanism show the necessity of clinical application of multiple antitumor technologies.

The correlation between each component of immune microenvironment and immunotherapy resistance is summarized in Table 2.

**TABLE 2:** Relationship between various components in tumor microenvironment and resistance to immunotherapy (PD‐1/PD‐L1 monoclonal antibody therapy)

Classes	Subclass	Functions	Resistance mechanism of immunotherapy	Prognostic predictor potential	Potential for joint application of ICB
TIL	CD4 + T Cell	Specific identification and kill tumor, determine the effect of antitumor immunity	Teff tumor invasion is reduced, and tumor cells produce immune evasion, which severely weakens the immunotherapeutic effect	Decreased density is significantly associated with the RFS.	Combined with chemokines and cytokines to promote tumor infiltration of effector T cells
	CD8 + T Cell				
	NK Cell				
	B Cell				
Immunosuppressive cell	Treg	Tumor progression is promoted by the release of immunosuppressive molecules such as TGF‐β, IL‐35, IL‐10, and depleted IL‐2, which inhibit or downregulate the induction and proliferation of effector T cells	Tumors can induce Treg cell production, promote CD8 + T cell failure, and develop drug resistance	The decrease in CD8 + T/Tregs ratio can be used as a negative predictor of anti‐PD‐1 monoclonal antibody efficacy	Combined with immune‐related factors to reduce immunosuppressive cells in TME
	MDSCs	It can inhibit effector T‐cell response and induce Tregs, promoting tumor angiogenesis, invasion, and metastasis	Immune tolerance is exerted in tumor microenvironment by the following factors: IDO, ARG1, ROS, il‐10, iNOS, cox‐2, and NO, etc	The level of MDSCs in TME was negatively correlated with prognosis	
	TAM（M2）	The immune escape of tumor cells can be mediated by PD‐1, and tumor invasion and metastasis can be promoted by angiogenesis and extracellular matrix remodeling	Immune escape of tumor cells is mediated by PD‐1	High levels of TAMs are associated with poor prognosis	Blocking the macrophage colony‐stimulating growth factor‐1 receptor (csf‐1r), reducing the frequency of TAMs, can increase the production of IFN, and improve the response of tumor cells to drugs. Csf‐1r blocker combined with PD‐1 or CTLA‐4 antibody, and then combined with gemcitabine, the treatment effect is better
Immune‐related factor	TGF‐β	Stimulating Tregs produces immunosuppressive effects	It can promote upregulation of tumor PD‐L1 pd‐l2 expression and recruitment of MDSCs, and promote antitumor immune resistance	Elevation is associated with poor prognosis in a variety of tumors	Inhibition of both TGF‐β and PD‐L1 receptors can reduce the tumor phenotype, improve survival and tumor development
	CCL2, CCL5, CCL7, and CXCL8 are associated with receptors CCR1 or CXCR2 on MDSCs, Treg, and m2‐type macrophages	MDSCs and Tregs can be transported to the tumor microenvironment	Resistance to PD‐1/PD‐L1 blockers was mediated by inhibiting Teff function		Chemokine receptor inhibitors can prevent the immune escape of tumor cells and improve the antitumor response of T cells
	CXCL12‐CXCR4 axis	Causes the polarization of the immune tolerance of Treg, M2, and N2 neutrophils	Manipulate the polarization and transport of immune cells to produce immunotherapeutic resistance		CXCL12‐CXCR4 axis inhibitor can be combined with PD‐1/PD‐L1 immunotherapy
	CXCL9‐CXCR3 axis	To enhance the functional activity of CD8 + T cells in tumor	Inhibition of CXCL9 and CXCL10 genes resulted in no response to anti‐PD‐1	CXCR3 ligands (CXCL9 and CXCL10) are positive indicators of anti‐PD‐1 response	Induction of CXCR3 ligand in non‐reactive tumors can restore the sensitivity of anti‐PD‐1, and the inhibition of CXCL9 and CXCL10 genes can be removed by the combination of epigenetic modulators
	ARG1 + EV	Immune cell suppression	Small EV can reduce T‐cell membranes containing ARG1 CD3 zeta and CD3 epsilon chain expression level. In vivo and in vitro inhibition of CD4 + and CD8 + T cells proliferation. ARG1 can be transported to other sites, such as draining lymph nodes, to promote immunosuppression and accelerate tumor progression.ARG1 + EV can be endocytosis by DC cells, thus inhibiting the stimulating effect of DC cells on T cells.	Peripheral blood detection of ARG + EV is negatively correlated with prognosis and has high clinical value	The ARG1 inhibitor reverses this inhibition
	PD‐L1 + exosome		Enhance Teff inhibition	The expression of exosome PD‐L1 is significantly correlated with ICB response, and can be dynamically measured	——
	CD38		It is an important catalytic enzyme in the adenosine production process, which can inhibit the proliferation and secretion of CD8 + T cells and the tumor killing effect through adenosine receptor 2A or 2B signaling cascade pathway	CD38 mRNA and protein levels were significantly elevated in drug‐resistant tumor cells	Combination of anti‐cd38 and anti‐PD‐1/ PD‐L1 therapy can improve the anti‐tumor immune response
Severe depletion of CD8 + T cells	Failing CD8 + T cells with moderate expression of PD‐1 and severely failing CD8 + T cells with high expression of PD‐1	CD8 + T‐cell failure, immunosuppression	Effector T‐cell failure, abnormal cytokines, and even loss, il‐2 production loss, significantly impaired IFN‐ γ production capacity, and severely impaired immune function	The ratio of failing CD8 + T cells with moderate expression of PD‐1 to severely failing CD8 + T cells with high expression of PD‐1 may be a key indicator to reverse resistance of PD‐1/ PD‐L1 antibody	The combination of immunocheckpoint inhibitors and t‐cell epigenomic engineering or epigenetic modulators may provide an opportunity for lasting efficacy of immunotherapy
Tumor transcriptome and epigenetic changes	Epithelial‐mesenchymal transformation‐related genes（AXL、 ROR2、WNT5A、LOXL2、TWIST2、TAGLN、FAP）	High expression, promote tumor	Produce tumor protein, promote tumor proliferation, and metastasis, Suppress immune response	Exon and transcriptome sequencing are of great value in the prediction of anti‐PD‐1 therapeutic response	Epigenetic changes in tumors are commonly seen in the methylation of CpG island in the promoter region of tumor suppressor genes. Histone acetylation, methylation, phosphorylation, ubiquitination, ADP ribosylation, and noncoding RNA can all affect the transcriptional activity of genes. Epigenetic groups have become new targets for individualized treatment of tumors. It can be used in combination with HDACi, BETi, IDHi, DOTi, EZH2 inhibitor, LSD1 inhibitor and DNMT inhibitor. respectively.
	Monocyte and macrophage chemokine genes（CCL2、CCL7、CCL8、CCL13）				
	Immunosuppressive factor gene（VEGFA、VEGFC、IL‐10）				
	Genes involved in damage, repair, and angiogenesis				
	TAA、HLA, and costimulatory molecules	Reduces or silences gene expression, evading recognition by the host immune system	Loss of immune‐stimulating antigen, Unable to produce an immune response		
	miRNA	Different members of the mir‐8 family (mir‐200a b and c) can also target the inhibition of the expression of the cancer PD‐L1 gene, resulting in increased CD8 + T cell activation and tumor immune surveillance	Mir‐8 transcriptional inhibition dissolves the inhibition of miRNA on the expression of PD‐L1 in tumor cells, leading to the failure of CD8 + T cells. Downregulation of mir‐8 family may be related to the resistance of PD‐1/PD‐L1 monoclonal antibody		
	LSD1	By increasing the level of H3K4me2 in the proximal promoter region, the expression of chemokines such as CCL5, CXCL9, and CXCL10 was inhibited, and CD8 + T lymphocytes were prevented from migrating to the tumor microenvironment	Lack of immune effector cells		
	EZH2	Mediates trimethylation of lysine 27 on histone 3 (H3K27me3) to induce chromatin compaction and transcriptional inhibition of target genes.	Overexpression is associated with an ineffective t‐cell response		
	DNMTs	Tumor suppressor gene promoters are prevented from expression in tumors by hypermethylation.	Tumor suppressor protein expression disorder		
PD‐L1 expression level	The mechanism affecting PD‐L1 expression involves induction of JAK/STAT signal, loss of PTEN, mutation of PI3K and/or AKT, EGFR mutation, overexpression of MYC, frequent amplification of chromosome 9p24.1 region, increase of PD‐L1 transcription level, etc Transcription factors ap‐1 and YY1 also have significant effects on the expression of PD‐L1.		Differences in the expression of PD‐L1 may induce different immune responses or tolerance to immunotherapy	The establishment of a comprehensive model based on the expression of tumor PD‐L1 and the characteristics of immune cell infiltration can better predict which patients can benefit from anti‐PD‐1/ PD‐L1 monoclonal antibody therapy	
Intestinal flora	Probiotics such as bifidobacterium	Metabolic changes induced by the microbiome regulate changes in TME and promote immune system function.	The imbalance of flora prevented the maturation of DC cells and the secretion of il‐12 factor, and the decrease in CD8 + T and CCR9 + CXCR3+CD4 + T cells in TME	Imbalance of intestinal flora can lead to reduced efficacy of ICBs (increased drug resistance)	The role and possible mechanisms of combined therapy as ICBs are still under investigation.
TLS	TLS has all the characteristics of normal lymph node formation, can produce anti‐tumor immune response, can promote T‐cell initiation, B‐cell activation and plasmocytic differentiation, is a precise factory for producing antibodies, and promotes increased cell infiltration of CD3+, CD8+, CD20 + and decreased cell infiltration of Foxp3 + and CD68 + in the tumor		The decrease in TLS number and low maturity leads to the suppression of tumor immune function	.The number and maturity of TLS in tumors may also be good biomarkers for the prognosis of various tumors	Immunoregulatory therapy targeted by TLS may be an effective potential strategy for immune‐mediated tumor suppression

## 4 ABNORMAL SIGNALING PATHWAY TRANSDUCTION AND RELATED PROTEIN GENE MUTATION IN TUMOR CELLS

Abnormal cell signal transduction is the core factor of immunotherapy resistance, including PI3K/AKT pathway, WNT/β‐catenin pathway, JAK/STAT/IFN‐γ pathway, and MAPK pathway.

### 4.1 IFN‐γ inactivation of the signaling pathway (JAK1/2/STAT/IRF‐1)

IFN‐γ regulates the immune response by regulating the expression of immune checkpoint proteins and the concentration of various chemokines. There are abundant high‐frequency mutations of proteins in the INF‐γ signaling pathway in tumor cells of immunocheckpoint‐resistant patients, such as IFN‐γ receptor 1 and 2, JAK1/2, and IRF‐1 (Interferon regulator factors 1). The whole‐exon sequencing technique was used to analyze and compare the whole‐genome sequence of tumor cells before and after the treatment of Pbolizumab. A homozygous incapacitated mutation of JAK1 and JAK2 was found in patients with recurrence. JAK2‐mutated tumor cells can also be recognized by CD8^+^T and produce IFN‐γ, but the JAK2/STAT/IRF1 signaling pathway cannot be activated by IFN‐γ, so it cannot upregulate the expression of tumor‐related antigen processing transporters, MHC‐Ⅰ, PD‐L1, and other genes downstream of the pathway, resulting in reduced killing effect of IFN on JAK2‐mutated cells. JAK1‐mutated cells were not sensitive to IFN‐α/β/γ. The tumor cells with JAK1/JAK2 gene mutation were not sensitive to the killing effect of IFN, and the expression of PD‐L1 was downregulated, making the tumor cells resistant to PD‐1/PD‐L1 monoclonal antibody.^138^, ^139^ Also found in the study of MHCⅠ important structure β2‐MG coding gene deletion mutation, the MHCⅠ heavy chain lost outside membrane positioning function, cannot present tumor specific antigen to T cells, is the important mechanism of the immune treatment of acquired drug resistance.^12^, ^140^

### 4.2 Mutation of the EGFR/ALK (Anapastic lymphoma kinase) signaling pathway

EGFR‐activated mutations are closely related to PD‐L1 expression and immune cell distribution in tumors.^141^, ^142^ Mutagenesis leads to a variety of resistance mechanisms that have been identified, including activation of the c‐MET (Mesenchymal‐epithelial transition factor) signaling pathway,^143^, ^144^ MET amplification, overexpression of HGF (Hepatocyte growth factor) and MET, amplification of HER2 (human epidermal growth factor receptor 2),^145^ EGFR, C797S, L792H, and G796R were mutated.^146^ The effect of anti‐PD‐1/PD‐L1 monoclonal antibody in NSCLC patients with epidermal growth factor/anaplastic lymphoma kinase (EGFR/ALK) mutation was poor, which was related to the simultaneous reduction in T effector lymphocytes in the tumor body under the influence of the mutation.^147^, ^148^, ^149^, ^150^ Therefore, it can also be considered to be related to the mechanism of EGFR‐TKI (Tyrosine Kinase Inhibitor) resistance in tumor cells. By observing the effects of HGF, c‐MET amplification, and EGFR‐T790M tumor on PD‐L1 expression and immune escape ability before and after EGFR‐TKIS drug resistance, the regulation mechanism of PD‐L1 in different drug‐resistant subtypes was discussed. It was found that HGF induced the expression of PD‐L1 in EGFR‐mutated NSCLC cells and regulated the proliferation and cytotoxicity of T lymphocytes: HGF not only activates the c‐MET signaling pathway in lung adenocarcinoma and induces drug resistance in EGFR‐TKIS^151^, ^152^ but also promotes the transcription of endogenous c‐MET genes.^153^ It is suggested that HGF may promote the immune escape of tumor cells through overexpression of PD‐L1 in EGFR‐TKI‐resistant NSCLC cells; HGF also induces PD‐L1 expression in NSCLC cells by activating PI3K/AKT, MAPK, and AP‐1. In EGFR‐TKIS‐resistant NSCLC cells, the PI3K/AKT and MAPK signaling pathways are involved in the upregulation of PD‐L1 induced by c‐MET amplification; EGFR‐T790M mutation upregulated PD‐L1 expression through the PI3K/AKT MAPK and NF‐kappa B (Nuclear factor kappa‐B, NF‐κB) signaling pathways. In tumors with normal distribution of TIL, the cytotoxic effect of T lymphocytes in vivo can be restored by downregulating the expression of PD‐L1 in EGFR‐TKI drug‐resistant lung cancer. Thus, acquired EGFR‐TKIs resistance promotes the immune escape of lung cancer by upregulating the expression of PD‐L1. PI3K/AKT, MAPK, NF‐Kappa B signaling pathway, and AP‐1 participate in the upregulation of PD‐L1 induced by different EGFR‐TKI resistance mechanisms.^154^ Other studies have also confirmed that EGFR/ALK mutations in NSCLC models upregulate PD‐L1 expression by activating the PI3K‐AKT, MEK (Mitogen‐activated protein kinase kinase)/ERK(Extracellular signal‐regulated kinase) pathway.^155^ And also produce or recruit more inhibitory cytokines and immune cells.^156^ Studies have shown that although the positive rate of PD‐L1 in tumor specimens with EGFR/ALK mutations is high, the ratio of high levels of CD8^+^TILs is very low,^149^ which limits the antitumor effect of anti‐PD‐1/PD‐L1 monoclonal antibody. The treatment of effective EGFR/ALK tyrosine kinase inhibitors will weaken the expression of PD‐L1 and remove the functional inhibition of CD8^+^T cells. When tumors are resistant to both tyrosine kinase inhibitors and PD‐1/PD‐L1 inhibitors, it is necessary to introduce other combination therapies, such as chemoradiotherapy with immunochemokines. It may effectively prolong the patient's clinical progression‐free survival, change the drug tolerance, and improve the prognosis.

### 4.3 WNT/β‐catenin signaling pathway

β‐catenin is the downstream protein of WNT signaling pathway in tumor‐related signaling pathway. After nuclear translocation, it binds to TCF (T‐cell factor) to directly mediate gene expression and promote cell growth and proliferation. Activation of WNT/β‐catenin signaling pathway in melanoma tissues was associated with loss of T‐cell gene expression characteristics.^157^ In the melanoma mouse model, β‐catenin activation reduced the expression of tumor CCL4 gene, and dendritic cells could not be recruited by the tumor microenvironment, thus, reducing the infiltration of T cells in the tumor. In this study, the combination of anti‐CTLA‐4 and anti‐PD‐1 monoclonal antibody significantly delayed tumor growth in control mice, but was not effective in β‐catenin–activated mice. Activation of the WNT/β‐catenin signaling pathway in CRC (Colorectal cancer) is closely related to CD8^+^T‐cell penetration. Activation of β‐catenin in CRC tumors can also significantly reduce the infiltration of CD8^+^T cells. It has been found that inhibition of the WNT/ β‐catenin pathway with an inhibitor (ICG‐001) can downregulate the expression of transcription factor ATF3, enhance the chemokine CCL4 in TME, and recruit CD103^+^DC cells. The mechanism of action is consistent with that of melanoma.^158^ Therefore, activation of the above carcinogenic signaling pathway can induce immune tolerance and is associated with resistance to PD‐1/PD‐L1 monoclonal antibody. The efficacy of anti‐PD‐1/PD‐L1 monoclonal antibody can be enhanced by the combination of related target inhibitors. Combined application of inhibitors of the WNT/β‐catenin signaling pathway with immunocheckpoint blockers may be a more effective clinical treatment for sensitive cancers. The combination of PD‐1 antibody and biological nanoparticles of β‐catenin siRNA has been effective in animal experiments, providing an effective strategy for clinical cancer treatment.^159^, ^160^

The WNT5a/β‐catenin pathway also regulates functional tolerance of DCs in the tumor microenvironment. Evidence suggests that tolerance of DCs in TME affects Teff activation and proliferation, promoting immune escape. The mechanism study found that WNT5a/‐βcatenin‐PPAR‐g (Peroxidase proliferation activation receptor‐g) signaling pathway was activated by paracrine in melanoma, upregulated the expression of CPT1A (Carnitine palmitotransferase‐1a) fatty acid transporter protein, which drives FAO (Fatty acid oxidation) in DCs, the change in FAO increased the protoporphyrin IX cogroup of IDO (Indoleamine 2, 3‐dioxidase‐1) and inhibited the expression of IL‐6 and IL‐12 cytokines, ultimately leads to the enhancement of IDO activity, the production of regulatory T cells, and the inhibition of Teff function; blocking this pathway can enhance antimelanoma immunity, enhance anti‐PD‐1 antibody immunotherapy activity, and inhibit disease progression. This mechanism hints at the role of tumor‐mediated metabolic reprogramming of local DC in immune avoidance and resistance to immunotherapy.^161^

The WNT/β‐catenin pathway inhibits tumor immunity through DC and produces resistance to PD‐1/PD‐L1 antibodies. Based on the above mechanism, WNT pathway, PPAR, FAO, IDO, and DC chemokines may become important targets of anti‐PD‐1/PD‐L1 monoclonal antibody resistance. However, this cascade relationship is affected by more other regulatory factors, such as the regulation of IL‐4 on PPAR, forming an intricate regulatory network. It is difficult for the regulation of a single target to completely eliminate drug resistance. Therefore, more effective treatment methods need to be explored.

### 4.4 PI3K‐AKT‐mTOR signaling pathway

The PI3K/AKT/mTOR signaling pathway regulates a variety of cellular processes, including apoptosis, proliferation, movement, metabolism, and cytokine expression. It is also related to the occurrence and development of tumors, and is one of the important mechanisms of the primary resistance mechanism of PD‐1/PD‐L1 antibody. Since the PI3K/AKT/mTOR pathway plays an important role in controlling a variety of intracellular processes, it is also regulated by many negative regulators to prevent abnormal activation. Lipid phosphatase PTEN is a class of tumor suppressor that can inhibit the activity of PI3K. PTEN deletion or mutation‐mediated PI3K/AKT activation and PD‐1/PD‐L1 resistance have been observed in many tumor types. The expression of PTEN is not only controlled by heterozygous or homozygous deletion but also by many different molecular mechanisms, including epigenetic silencing, post‐transcriptional and post‐translational modifications, and protein‐protein interactions. One of the main mechanisms for constitutive activation of the PI3K/AKT/mTOR pathway is the missing phosphatase and tensin homologous in the expression of chromosome 10 (*PTEN*).^162^ PTEN is the PIP3 3‐phosphatase encoded by the *PTEN* gene on chromosome 10q23^163^ ^.^ Since PTEN is involved in the control of a range of processes, including tumor growth and spread, metabolism, aging, and EMT, its downregulation plays a key role in the progression of many types of cancer. The loss of PTEN leads to changes in the PI3K/AKT/mTOR pathway that affect cell energy metabolism, and the metabolic reprogramming of cancer cells is another important feature of cancer. PTEN inactivation increases glucose uptake by transport of GLUT4 (glucose transporter 4) on the plasma membrane.^164^ The loss of PTEN function also leads to the activation of 4EBP1 (4E Binding Protein 1) and p70S6 (p70 Ribosomal Protein S6 Kinase) kinase, which participate in the protein synthesis process.^165^ Besides, PTEN is involved in cell migration and cell senescence. In gastric and lung cancer, downregulation of PTEN expression is associated with activation of FAK (Focal adhesion kinase), resulting in increased cell viability.^166^, ^167^ Loss of apical‐basal polarity promotes EMT and enhances tumor cell migration^167^, ^168^ ^.^ The above factors have significant negative effects on tumor immunotherapy. Some evidence has highlighted the correlation between the deletion of PTEN and resistance to immunotherapy, suggesting that the deletion of PTEN gene may be one of the mechanisms driving drug resistance in tumors to PD‐1/PD‐L1 inhibition.^169^ These results suggest that the ability of PTEN to modulate the tumor microenvironment may be the result of altered cytokine patterns secreted by the tumor stroma. At the molecular level, loss of PTEN in tumor cells leads to a significant downregulation of SHP‐2 (Src homology‐2‐containing protein tyrosine phosphatase 2), a negative regulator of the JAK/STAT3 pathway. Studies have shown that SHP‐2 protein deficiency can maintain tumor growth by promoting activation of the JAK/STAT3 pathway. This association was further demonstrated in other types of tumors.^170^, ^171^ In addition, the loss of PTEN in melanoma, prostate cancer, and glioblastoma tumors is associated with decreased T‐cell function, increased VEGF production, and the release of anti‐inflammatory cytokines, leading to an increase in noninflammatory tumors and changes in tumor microenvironment. The effect of PTEN on PD‐1/PD‐L1 monoclonal antibody response is not only limited to the change in tumor microenvironment but also related to the ability of PTEN to regulate PD‐L1 level. Studies have found that the absence of PTEN or constitutive expression of the PI3K/AKT pathway can regulate PD‐L1 expression in some tumor types in an IFN‐γ‐dependent and ‐independent manner. Constitutive activation of PI3K signal increased PD‐L1, at least partly due to the change in PD‐L1 mRNA level.^79^ In colorectal cancer cell lines, the silencing of PTEN leads to the increase in PD‐L1 at the membrane level, while PD‐L1 mRNA does not, indicating that the loss of PTEN may contribute to the stabilization of PD‐L1 protein.^172^ The correlation between PTEN and PD‐L1 in tumors was also reversed. Hlaing and colleagues found that PTEN expression was positively correlated with PD‐L1 level, which was different from the previously reported data on other types of tumors.^173^, ^174^ It is speculated that the expression of PD‐L1 is regulated by various intracellular signaling pathways (mainly tumor type signaling pathways), such as RAS/RAF/MEK, PI3K/AKT/mTOR, JAK, STAT, or IFN‐γ signaling, in which IFN‐γ is released by immune cells in the tumor microenvironment. High PTEN expression in patients with high PD‐L1 may be due to cross‐talk of other pathways in these tumor types, such as high IFN‐γ TME status. In mouse experiments, when treated with selective inhibition of PI3K, the therapeutic effect of PD‐1/PD‐L1 antibody or CTLA‐4 monoclonal antibody was enhanced.^175^ Further clinical studies are needed to determine whether PI3K/AKT inhibitors can reverse the resistance process of immunocheckpoint inhibitors.

### 4.5 RAS/MAPK pathway

In LUAC (Lung adenocarcinoma), the carcinogenic *KRAS* mutation leads to the loss of *STK11(LKB1)*, thereby recruiting neutrophils with T‐cell inhibitory effects, resulting in reduced T‐cell infiltration. The absence of *STK11* is the main driver of resistance to PD‐1 in *KRAS* mutant tumors.^176^ Furthermore, *KRAS* mutation inhibits the expression of IRF2, which in turn directly inhibits the expression of CXCL3. CXCL3 binds to CXCR2 on MDSCs from bone marrow to promote its migration to the tumor microenvironment. The anti‐PD‐1 resistance of KRAS‐mutated tumors can be overcome by forcing IRF2 expression or inhibiting CXCR2. High IRF2 expression in CRC is associated with increased response to PD‐1 immunotherapy. The KRAS* ‐IRF2‐CXCL3‐CXCR2 axis provides a theoretical basis for CRC patient selection and combination therapy to enhance the effectiveness of ICB therapy.^177^ In human melanoma samples, inhibition of RAS/MAPK pathway can promote the activation and infiltration of CD8^+^ T cells, induce the expression of tumor antigen, and enhance antitumor immunity.^178^ Acquired resistance to RAS/MAPK inhibitors is associated with reduced CD8^+^ T‐cell failure and antigen expression loss in tumor T cells.^179^, ^180^ Experimental results showed that the treatment strategy combined with PD‐1 antibody and MEK blocker enhanced the infiltration of immune cells in the tumor and improved the outcome of the tumor.^181^

In summary, drug resistance of PD‐1/PD‐L1 blockers is closely related to abnormal changes in complex signal transduction pathways in tumor cells. Various signal molecules and corresponding genes interact, interlacing restrict or promote, forming a multiple regulatory network, and its overall mechanism is still difficult to be fully explained at the current research level. However, immunotherapy tolerance can eventually be induced by reduced tumor infiltration of immune cells, depletion of effector T cells, increased recruitment of immunosuppressive cells, or release of immunosuppressive cytokines. Because there are many mechanisms involved in drug resistance and their targets are complex, the effectiveness of existing combined regiments against drug resistance remains to be verified clinically. However, on the basis of the existing combined drug regimen, more targeted combined drug regimen seems to be more helpful to solve the drug resistance of PD‐1/PD‐L1 monoclonal antibody theoretically. Due to the heterogeneity of tumors, clinical laboratory analysis can only reflect the variation (mutation) characteristics of the dominant tumor cell population. The combination therapy for the mutant target may inhibit or destroy the sensitive dominant tumor cell population in the short term, but the nondominant tumor cells that are not sensitive to the combination therapy may accelerate their proliferation and replace the original dominant cell population due to the release of living space caused by the above treatment, which may lead to drug resistance again. Such circulation may be an important reason why cancer treatment is not effective. Therefore, the new strategy of repositioning the tumor combination therapy is more important for the long‐term expected efficacy of the tumor.

## 5 PD‐1/PD‐L1 RESISTANCE AND CELL METABOLISM AND METABOLITES

In tumors, the lack of nutrients can seriously affect cell proliferation, survival, and function. Tumor‐infiltrating T cells get trapped in a rich metabolic network built up in harsh microenvironments and are forced to face relentless nutritional competition. Cancer cells can express a variety of enzymes that cause T cells to lose key substrates and produce immunosuppressive metabolites. Metabolic changes run through the whole life cycle of T cells and provide the necessary energy supply and survival resources for T cells to help them meet the emerging needs.^182^ In the tumor microenvironment, tumor cells occupy most available resources in a powerful competitive way, which depletes the nutritional supply of immune cells and inhibits their functions. The PD‐1/PD‐L1 pathway influences TIL cell metabolism, alters the expression of genes in related metabolic signaling pathways, and induces drug resistance.

### 5.1 Glucose metabolism in tumor TME

Glycolysis is a key metabolic axis to maintain immune balance. The glucose metabolism of naive or inactive T cells is mainly dependent on OXPHOS (Oxidative phosphorylation) to increase energy. However, once activated, the T cells switch to aerobic glycolysis mode. In this rather inefficient process, each glucose molecule produces only two ATP, with a low ATP/glucose ratio. However, aerobic glycolysis provides more basic conditions for anabolism, and is an essential process for T cells to obtain multiple effector functions, eg, IL‐2, IFN‐γ, and so on. During the production of IFN‐γ, GAPDH (Glyceraldehyde‐3‐Phosphate Dehydrogenase) can reduce the block of IFN‐γ mRNA translation and accelerate its synthesis.^183^ Metabolic conversion during T‐cell activation is controlled by a large number of signaling pathways and transcription factors. TCR involvement, costimulation, and cytokine signaling promote glycolysis by upregulating the expression of nutrient transporters (such as the GLUT1 glucose input) and by activating the mTOR complex, a central metabolic regulatory target.^184^, ^185^ The activation of mTOR leads to the rapid upregulation of GLUT1 expression and the increase in its transport to plasma membrane, leading to rapid reinforcement of glucose input. However, in the tumor microenvironment, tumor cells are more competitive than T cells in glucose uptake and aerobic glycolysis, which can inhibit the functional effects of T cells and thus evade immune killing. In terms of mechanism, Ho et al demonstrated that TCR‐induced Ca2^+^ flux in T cells is critical for stabilizing glucose metabolism. Extracellular glucose promotes glycolysis to produce phosphoenolpyruvate accumulation, inhibits Ca2^+^ from cytoplasmic chelation into ER, and thus maintains activation‐induced Ca2^+^ flux and T‐cell effector function. On the contrary, increased hexokinase 2 expression in tumor microenvironment leads to inhibition of extracellular glycolysis, decreased Ca2^+^ flux in T cells, decreased immune cell effector function, and promoted tumor evasion from T‐cell–mediated immune monitoring.^186^ In terms of mechanism, other studies have suggested that PD‐L1 signal in tumor cells can promote the ability of glycolysis in tumor cells by activating AKT/mTOR pathway. Blocking therapy of PD‐L1 can reduce the glycolysis rate by triggering the internalization of PD‐L1, restore the glucose level in the microenvironment, and hinder the progress of the tumor.^187^ However, in RCC (Renal cell carcinoma) tumor cells resistant to PD‐1 monoclonal antibody, metabolic changes that meet the energy demand for rapid cell proliferation are shown, such as significantly upregulated levels of hypoxia adaptation factors, glucosaldehyde acidification, and overexpression of nutrient transportation‐related molecules. The overexpression of metabolic genes reflects that the tumor restricts the reactivity of tumor‐specific infiltrating T cells by competing for important nutrients such as glucose in the tumor microenvironment and mediates the development of drug resistance. In conclusion, regardless of the mechanism, the advantage of tumor cells in glucose occupation or consumption is far greater than that of immune cells, resulting in the lack of energy and necessary synthesis supply of immune cells. Simply blocking the PD‐1/PD‐L1 checkpoint cannot solve the resource deficiency state of immune cells, so they cannot play their normal functions due to exhaustion. Further understanding of the markers of the underlying mechanisms of this tumor may reveal new therapeutic targets for combination therapies based on the blocking of the PD‐1 pathway, as well as the selection of useful markers for patients most likely to respond to these therapies.

### 5.2 Amino acid metabolism in tumor microenvironment

Trp (Tryptophan) is an essential amino acid for the proliferation and differentiation of human T lymphocytes, it is an important nutrient to maintain its normal physiological function. IDO is the key enzyme in the first rate‐limiting step on the Kyn (kynurenine) pathway of Trp metabolism outside the liver, which converts the essential amino acid L‐Trp into the main metabolite Kyn.^188^ IDO exists in two forms, IDO1 and IDO2, derived from two separate genes.^189^ Compared with IDO1, IDO2 is relatively weak in function and does not participate in systemic tryptophan metabolism, but its interaction in TME is unclear.^190^, ^191^ Increased IDO level can lead to tryptophan depletion, inhibit T‐cell Trp supply, induce G1 phase T‐cell cycle stagnation, affect the cell cycle of lymphocytes in the tumor microenvironment, and be sensitive to apoptosis.^192^, ^193^, ^194^, ^195^ In addition, metabolites Kyn, 3‐HAA (3‐hydroxyanthranilic acid), and QA (Quinolinic acid) along the Kyn pathway can activate the aromatic hydrocarbon receptor Ah R (Aryl hydrocarbon receptor). A large number of studies have confirmed that Ah R promotes tumor development and participates in immune escape.^196^, ^197^ Many tumor types show an overexpression of IDO.^198^ The mechanisms of IDO expression increase in tumor tissues mainly include the absence of tumor suppressor gene *Bin1* (Bridging integron‐1),^199^ the expression of tumor microenvironment interferon,^200^ and COX‐2 promoting IDO expression,^201^ etc The overexpression of IDO is largely related to the poor prognosis of tumor.^202^ More and more evidences show that serum Kyn/Trp ratio is related to immunotherapy response and survival prognosis, and drug resistance is an important reason. Excessive increase of IDO activity may cause cell cycle, gene repair, and immune dysfunction, resulting in resistance to various tumor therapeutic drugs including immunocheckpoint inhibitors.^203^, ^204^, ^205^, ^206^, ^207^ In the mechanism of IDO increase, IFN‐γ is closely related to ICB. ICB applications can increase the generation of IFN‐γ.^208^, ^209^ In some tumor cells, an increase in IFN‐γ has been demonstrated to induce an increase in IDO production,^210^ further promotes resistance to monoclonal antibody CTLA‐4 or PD‐1 treatment, leading to immune escape, which can be overcome by IDO inhibitors. To further study the role of tryptophan metabolism abnormality caused by IDO in the pathogenesis and treatment of tumors, and to explore appropriate targets will provide new ideas for the treatment of tumors, and bring good news to tumor patients.

In addition to tryptophan, other amino acids and their metabolic enzymes also affect local immunity in TME. Arginine has the function of promoting immune system in the body; it can significantly stimulate T lymphocyte proliferation response. With the participation of arginase, the semi‐essential L‐arginine can be catalyzed to degrade to L‐ornithine and urea. Consumption of L‐arginine in the tumor microenvironment prevented the progression of the T‐cell cycle and inhibited the production of IFN‐γ. Arginase also downregulates CD3 ζ and ε chain expression associated with T‐cell receptors. This is a key component of the TCR signaling complex, which impairs T‐cell function. There are two subtypes of argininase (ARG1 and ARG2) that catalyze the same biochemical reactions, but they differ in subcellular localization, expression, and regulation. ARG1 is a cytoplasmic protein and ARG2 is mainly located in mitochondria. High arginase levels, ARG1 or ARG2, are present in a variety of cancer types, including breast cancer, NSCLC, head and neck squamous cell cancer, kidney cancer, colorectal cancer, skin cancer, and cervical cancer. Arginase is mainly produced by MDSCs highly enriched in TME, and ARG1‐expressing MDSCs play a role in changing T‐cell response in cancer patients, enhancing immunosuppressive cell function, influencing immune network regulation, and influencing ICB efficacy.

In short, the normal physiological function of the body's immune cells depends on the normal and orderly metabolism of essential amino acids. Abnormal metabolic substrates, metabolic enzymes, and metabolites can seriously affect the immune function and lead to the failure of immune cells. In order to meet the needs of rapid proliferation and infiltration of tumor cells, the original amino acid metabolism balance of immune cells was destroyed under the combined action of multiple factors in TME, and the immune function was impaired. At this point, PD‐1/PD‐L1 blockers cannot reverse cell failure, resulting in tolerance. Therefore, in clinical treatment, it is necessary to combine IDO, ARG, and other amino acid metabolism enzyme inhibitors according to the microenvironmental conditions with abnormal amino acid metabolism, so as to remove the resistance of PD‐1/PD‐L1 blocker and improve the curative effect.

### 5.3 Immunosuppression of adenosine production in tumor microenvironment promotes drug resistance of PD‐1/PD‐L1 blockers

Extracellular adenosine is an immunosuppressive metabolite in tumor microenvironment, which is abundant in the extracellular components of TME. The growth and necrosis of the tumor resulted in a large amount of extracellular ATP (Adenosine triphosphate), and the enzymes CD39 and CD73 synergistically ATP to form adenosine.^211^ By activating AR (Adenosine receptor) to participate in the regulation of a variety of physiological and pathological processes in the body, these cascade steps can eventually promote the transformation of tumor local tissues from a pro‐inflammatory response to an anti‐inflammatory response, adversely affecting cytotoxic CD8^+^ T cells NK cells and dendritic cells.^212^, ^213^ In addition, there is another adenosine production pathway, CD38 catalyzes the production of ADPR (Adenosine diphosphate ribose) or cADPR (Cyclic ADPR) using NAD^+^ (Nicotinamide adenine dinucleotide) as a substrate,^77^ and ADPR catalyzed by CD203 enzyme to generate adenosine monophosphate, which was then dephosphorylated by CD73 to generate adenosine, providing a secondary pathway to bypass CD39 to generate extracellular adenosine. Therefore, under the action of CD38, the tumor microenvironment will eventually reduce extracellular NAD^+^, change the cascade of calcium signals, and produce immunosuppressive adenosine. There are four known subtypes of adenosine receptors: A1R, A2aR, A2bR, and A3R, all of which are GPCR.^214^ A2aR and A2bR are closely related to the occurrence, development, metastasis, and immune escape of tumors, and are also considered as potential tumor therapeutic targets.^215^ It can promote tumor cell proliferation,^216^, ^217^, ^218^ metastasis,^219^, ^220^ and change tumor microenvironment.^221^ The A2aR is the main expression subtype in most immune cells. In the tumor microenvironment, A2aR is overexpressed in all kinds of tumor cells and immune cell subsets, and is associated with many immunosuppressive factors. Most of A2aR highly expressed in immune cell subsets in tumor tissues reflects the promoting effect on tumor development and the inhibiting effect against tumor immunity.^222^ The immunosuppressive effect of A2aR is usually achieved by inhibiting effector T‐cell proliferation, cytokine production and cytotoxicity, and reducing chemotaxis^223^; provide immunosuppressive signals of NK cells and NKT cells; trigger activation of A2aR on Treg causes cell amplification and increases its immunomodulatory activity.^224^ FoxP3 is a key transcription factor in the immunosuppressive activity of Treg cells and can be induced by A2aR stimulation. The presence of A2aR stimulation in T‐cell activation significantly increased CD4^+^FoxP3^+^ cells. In addition to increasing the number of Treg cells, A2aR stimulation also enhanced the immune regulatory activity of Treg cells. A2bR was activated only under pathophysiological conditions (eg, in inflammatory TME) during an increase in adenosine concentration. A2bR activation inhibits isopentenization of GTPase Rap1B (Guanine 5′‐triphosphate Ras‐proximate‐1), leading to decreased intercellular adhesion mediated by Rap1B and promoting metastasis of cancer cells.^225^ In evaluating the efficacy and mechanism of A2aR inhibitor and anti‐PD‐1 monoclonal antibody combination, it was found that^226^: compared with monotherapy, this combination significantly reduced tumor metastasis and extended the lifespan of the mice. Therefore, the use of A2aR inhibitor in combination with anti‐PD‐1 monoclonal antibody in the treatment of extracellular adenosine‐induced immunosuppressive tumors (TME improvement) is worth further study.

In summary, the metabolites adenosine in TME binds to the corresponding receptors and inhibits the pharmacological action of PD‐1/PD‐L1 blocker, resulting in drug resistance effect. Immune effector cells lose the function of activation, proliferation and tumor chemotaxis, and are replaced by immunosuppressive cells, such as Treg and MDSC, etc Immune checkpoint blockers lose the basic conditions for producing antitumor immunity due to the lack of effective targets in TME. Therefore, in view of such drug resistance mechanism, the combination of ICB and inhibitors of related metabolic enzymes in adenosine production pathway or adenosine receptor inhibitors is expected to achieve long‐term clinical efficacy, and adenosine enrichment degree in tumors may also become a biomarker of immunoprecision therapy.

### 5.4 SK (Sphingosinol metabolic kinase) in tumor microenvironment

Cell membrane sphingomyelin derivatives Cer (Ceramide), Sp (Sphingosine), and S1P (Sphingosine 1‐phosphate) play important roles in the regulation of cell proliferation, survival, and apoptosis. S1P is a regulator of lymphocyte transport and differentiation under different pathophysiological conditions,^227^, ^228^ produced by SK (Sphingosine kinase), which catalyzes the phosphorylation of sphingosine to S1P. SK is a key enzyme that regulates the metabolic balance of Sp, SlP in cells. SK1 (SK subtype 1), encoded by the *SPHK1* (Sphingosine Kinase 1) gene, is overexpressed in many human tumors, including melanoma, resulting in increased S1P levels.^229^, ^230^ The SK1/S1P axis can regulate the different characteristics of cancer such as cell proliferation, cell death, metastasis, and angiogenesis.^231^, ^232^ SK1 overexpression has been described in many different cancer types, including lung cancer,^233^ gastric cancer,^234^, ^235^ breast cancer,^236^, ^237^ and glioblastoma.^238^ In a meta‐analysis of clinical studies, high expression of SK1 was associated with reduced survival in patients with various cancers.^239^ In melanoma patients treated with anti‐PD‐1, high expression of SK1 in tumor cells is associated with shorter survival. SK1 produces phospholipid‐like S1P, which functions by intracellular action or by binding to five cell surface S1PR1‐5 (G‐protein‐coupled receptor isoforms), which are expressed in both cancer cells and TME.^240^, ^241^ The SK1/S1P/S1PR axis can regulate the behavior of tumor cells and the composition of TME.^231^, ^242^ Studies have demonstrated that tumor SK1 plays a key role in the regulation of TIL components, leading to the accumulation of potent Tregs. Targeting SK to regulate sphingolipids metabolism can improve tumor immunotherapy response.^243^ ICB binding with SK1‐targeted inhibitors can significantly increase CD8^+^T/Treg ratio, supporting the important role of tumor SK1 in immune escape..Therefore, inhibition of SK1 may be an important strategy to enhance ICB response. Further studies found that S1PR1 signaling in T cells enhanced tumor invasion by Treg in a STAT3‐dependent manner, reduced CD8^+^TIL in TME, and increased breast cancer and melanoma growth in mouse models.^244^ Moreover, since S1P signals are translocated through SPNS2 (S1P transporter) in the blood and lymphatic system, the effect of targeting SPNS2 on the immune response has also been elucidated.^245^, ^246^ Loss of SPNS2 reduces the amount of metastatic melanoma aggregation in the lung, both at the systemic level and in a specific manner within the lymphatic endothelium, and is associated with increased infiltration of activated CD8^+^T cells and NK cells in the lung.^245^ Another analysis showed that in melanoma tumors, SK1 knockdown significantly reduced the production of various immunosuppressive cytokines, such as TGF‐β, IL‐10, CCL17, and CCL22, which was consistent with the explanation of the significant decrease in tumor infiltration of Treg. When SK1 was silenced, PGEs was significantly reduced, leading to a significant reduction in the production of PGE2 (Prostaglandin E2 synthase), and enhancing the therapeutic response of melanoma to PD‐1/PD‐L1 monoclonal antibody.^247^ The combination of ICB and SK1 antagonists may be an innovative anticancer therapy option.

The effect of sphinoline 1‐phosphate on Treg enrichment in tumors determines the efficacy of PD‐1/PD‐L1 blocker. The number and function of CD8^+^T cells in TME decreased, and their distribution was rare, and peripheral TLs were difficult to establish. At this point, immunocheckpoint inhibitors alone cannot activate immune cells in the local microenvironment of the tumor, showing clinical resistance. Abnormal changes in the distribution of SP, SK, S1P, SPNS2, S1PRs, downstream molecular targets (such as RAS, MAPK, PI3K, PLC, etc), corresponding nuclear transcription factors, immune cells, and related factors can affect the therapeutic effect of ICB to varying degrees. In the S1P regulatory network, intermolecular crosstalk makes functional regulation more complex, and a single molecular target has limited effect, which is also prone to drug resistance. In view of the pathological basis of such clinical immunotherapy tolerance, comprehensive treatment should be adopted to restore the function and number of tumor local effector T cells. Combined chemoradiotherapy or Oncolytic virus therapy destroys tumor microenvironmental structures, rational use of cytokines and chemokines can improve the number of DC and Teff/Treg ratio in tumors. Inhibit SK activity, block SPNS2 transport function or inhibit S1PRs to block downstream molecular activation, change TME non‐tumor cell composition, and active factor secretion. Combined with PD‐1/PD‐L1 blocker, CD8^+^T lymphocytes were activated to maintain long‐term local immune clearance.

### 5.5 UGT metabolic enzymes

UGT enzymes is the key enzyme of the metabolism of Ⅱ phase, catalytic uridine diphosphate glucuronic acid groups of glucuronic acid is transferred to a variety of endogenous and exogenous compounds, increase its polarity, is easy to be out of the body along with urine and bile.^248^ UGT enzymes not only participate in the metabolism of exogenous drugs but also participate in the metabolism of many endogenous substances such as bilirubin, short‐chain fatty acids, bile acids, and fat‐soluble vitamins, which is an important detoxification pathway in the body. The gene‐encoding UGT enzyme has 2 families (*UGT1, UGT2*) and 3 subfamilies (*UGT1A, UGT2,A* and *UGT2B*). The *UGT1A* gene cluster is located in chromosome 2q37 and has a total length of 200 KB. It is a very important member of the *UGT* gene family, encoding 9 UGT1A proteins, including UGT1A6 and UGT1A9. In the tumors of patients with advanced renal clear cell carcinoma, the expression level of UGT1A6 was also significantly increased, and the expression level of GUT1A6 in those who did not respond to PD‐1 blocking antibody Nivolumab was 288 times higher than that in the responders, and the expression levels of UGT1A1 and UGT1A3 were also significantly increased (5 times and 7.1 times higher than the responders, respectively).^249^ The expression of UGTs was influenced by endogenous metabolites, exogenous diet, environment, and drug factors. Tumor genomics studies have found that the increased expression of genes related to metabolic function in RCC is related to the use of anti‐PD‐L1 monoclonal antibody and produces resistance. UGT1A6 was overexpressed in PD‐L1^+^PD‐1 monoclonal‐resistant RCC. UGT1A6, whose main function is to promote the elimination of toxins and exogenous lipophilic chemicals, is a single highly expressed molecule related to the resistance of PD‐1 immunotherapy. In addition, the expression of other UGT1A family members and solute carriers constituting chemical defense was also upregulated, which may lead to the enhanced ability of UGT1A6 as a representative molecule to remove tumor cytotoxins, making it more conducive to the competition with the immune system.^249^ These studies suggest that new targets may be found in the study of tumor metabolic pathways. The combination of regulation of tumor cell metabolism and anti‐PD‐1 monoclonal antibody therapy can improve the tumor microenvironment and enhance the sensitivity of cancer cells to drugs. It is an optional treatment strategy and may reverse the therapeutic effect of drug resistance.

## 6 DISCUSSION

**FIGURE 3:**
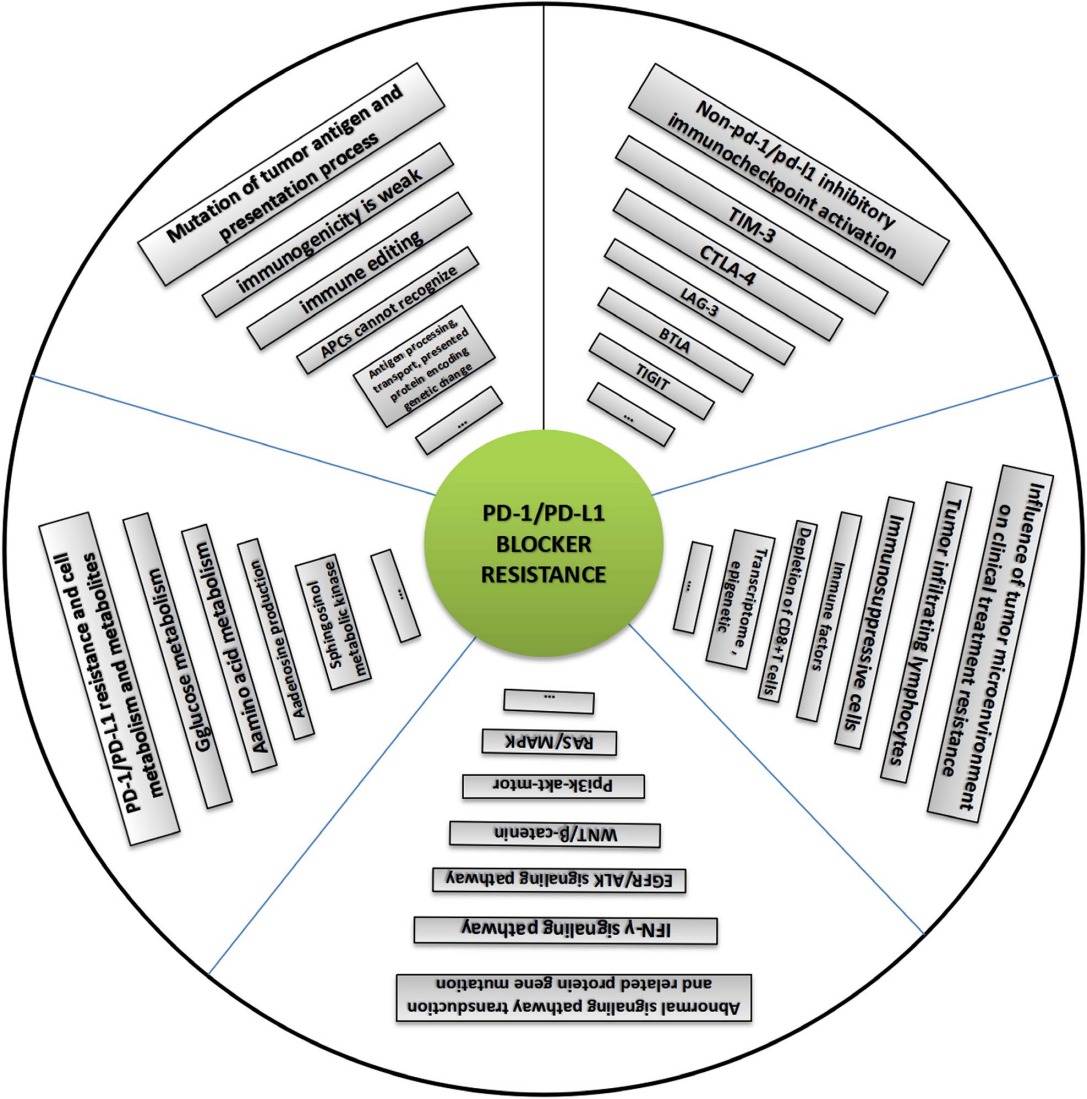
Summary of resistance factors of PD‐1/PD‐L1. Tumor resistance to PD‐1/PD‐L1 immunotherapy is mainly related to the influence of the five aspects in the figure, each of which can be subdivided into multiple factors to form an interwoven network of mutual influence. Combined multi‐target therapy can improve the tumor response rate, inhibit tumor development, and improve the survival rate of patients

The clinical application of PD‐1/PD‐L1 monoclonal antibody provides a new target and definite efficacy for tumor immunotherapy. However, due to primary or secondary drug resistance, the clinical beneficiaries are limited, and promotion is hindered. To explore the mechanism of immune checkpoint inhibitor resistance has become a hot research topic. Drug resistance is a common phenomenon in tumor therapy, especially for patients with specific target and single drug. The complex regulatory mechanism inside and outside the cell can change the drug blocking action, reach new signal transmission or metabolic balance, and continue to maintain the growth of tumor cells. The characteristics of tumor heterogeneity further enhance the intratumor cell and TME regulatory network complexity. Multiple factors interweave and cross‐talk, dynamic checks and balances, and affect the physiological and pathological regulation of tumor and immune cells. As shown in Figure 3. So far, it is difficult to find a universally applicable biomarker to evaluate the efficacy or drug resistance of PD‐1/PD‐L1 inhibitors, and it is also difficult to find a decisive link target to improve ICB resistance. It is of little clinical significance to simply search for and regulate drug resistance targets, and it can even produce drug resistance again soon, which cannot completely solve the ICB resistance situation, and thus cannot expand the clinical adaptive population of ICB preparations such as PD‐1/PD‐L1 monoclonal antibody. In summary, through the one‐by‐one analysis of the formation mechanism of drug resistance of PD‐1/PD‐L1 blockers in tumor therapy, it was found that they involved the comprehensive effects of multiple factors including genetics, epigenetics, tumor signaling pathway, cytokines, immunogenicity, antigen presentation, cell metabolism, exosomes, and tumor microenvironment. Clinical individualized precision therapy may still generate drug resistance through various network crosstalk in the later stage, which poses a challenge to explore clinical ICB resistance solutions. Since the exact mechanisms of primary and secondary drug resistance in tumor therapy have not been recognized, they may be related to the complexity and uncertainty of tumor formation. Existing studies have found that there are a large number of heterogeneous tumor cells, and there are also different heterogeneous tumor cells or stem cells in the same tumor tissue. Under the influence of different environmental factors, the initial tumor cells can mutate or differentiate into different subsets of branches and have the stem cell‐like cells that can be maintained by the subsets. Moreover, the differentiated subsets of cells can also dedifferentiate and possess the characteristics of stem cells under special external effects, jointly maintaining the development of the subsets. Therefore, it can be inferred that due to the limitations of living space and conditions, there is a fierce survival competition (free competition period) among the tumor cells in the early stage. At this time, tumor subsets of cells are interdependent with each other and compete for territory with normal tissues, forming a specific tumor microenvironment; internal mutual inhibition, competition for survival resources, and improvement of predatory ability also limit the rapid progress of tumors to a certain extent, so generally speaking, early tumor development cycle is long, slow growth. In the middle and late stage of tumor, due to the difference in competitive ability, the growth of some inferior subgroups is inhibited to different degrees, while the other dominant subgroups compete to the absolute survival conditions and space, and grow rapidly (monopolistic growth period), occupying the majority of tumor tissues and dominating tumor properties, biological characteristics, and development prognosis. It is further speculated that primary drug resistance may be related to the mechanism of drug resistance existing in the dominant growth subsets of cells, and the selection of therapeutic drugs should be considered in accordance with the main biological characteristics of the tumor, sensitivity biomarkers, gene mutation analysis, and other factors, so as to avoid the occurrence of primary drug resistance; in most cases, the dominant subgroups in tumors dominate the biological characteristics, and the existing clinical tumor individual analysis and diagnosis, the proposed treatment plan, and drug selection are more targeted at the dominant subgroups of cells, which are sensitive to treatment, while most of the inferior subgroups of cells may not be sensitive to treatment. Therefore, after treatment, sensitive tumor cells are inhibited or eliminated to varying degrees, the original TME balance is destroyed, and tumor mass can be shrunk or even clinically cured. At this time, the survival conditions and space of inferior subgroup cells were released in the remaining tumor or residual lesions. Under the pressure of multiple selection, such as drugs or immunity, new dominant cell subsets were selected after recompetition among inferior subsets of cells, and then migrated and colonized after rapid in situ growth or depolarization. The clinical manifestations were secondary drug resistance or recurrence and metastasis, which were generally resistant to the original treatment regimen. In this way, the existing drug treatment of tumors is doomed to a poor long‐term prognosis and difficult to cure. Based on the above hypothesis, the formulation of the current clinical treatment scheme for tumors should be improved from the perspective of thinking. In principle, drug use should not be focused solely on sensitive biomarkers or overly specific targets, but should be evaluated on this basis in combination with an evidence‐based approach based on tumor pathological types. Early combined use of antitumor drugs for blocking treatment, in order to achieve the synchronous inhibition and elimination of tumor multisubsets of cells, reduces drug resistance or recurrence and metastasis, and improves the long‐term efficacy.

Therefore, in terms of clinical treatment, how to choose combination therapy may have an important impact on tumor resistance and recurrence. At present, tumor treatment methods mainly include the following aspects, such as surgical resection, chemoradiotherapy, oncolytic virus, endocrine, molecular targeting, immune targeting, and immune checkpoint therapy, etc, and various treatments are further subdivided into more treatment methods. Because different treatment methods are applicable to different tumor range. For example, endocrine therapy has a clear therapeutic effect on breast cancer and prostate cancer, but may not be applicable to the treatment of other tumors. According to the range of tumor types that can be used clinically, we generally divide the tumor treatment methods into three types: broad, medium, and small. A broad range of therapeutic types, such as surgical treatment, radiotherapy, and chemotherapy, are beneficial to the vast majority of clinical treatments for all types of tumors and their subcellular groups. They belong to the traditional tumor treatment mode and can be used as basic treatment. The medium range of treatment types, such as endocrine therapy, immunotherapy, and oncolytic therapy, has therapeutic effects on a class of tumors with similar pathological mechanisms or subcellular populations within the tumor. It is not targeted at individuals, but can be used as appropriate to evaluate the comprehensive efficacy of a class of tumors based on evidence‐based medicine. Small‐range therapy types refer to individualized precision therapy. Targeted small molecules, antibody‐targeted drugs, immune cells embedded with specific antigen receptors, or engineered recombinant oncolytic virus therapy is selected according to the individual characteristics of tumor biology, which is highly targeted at the dominant growth cell subsets that dominate the progression in tumor tissues. Each of the above three types of treatment has its own advantages. It can better optimize the clinical treatment plan by synergistic complementation and rational application. For example, for tumor patients with drug resistance to PD‐1/PD‐L1 blockers, radiotherapy and chemotherapy, immunotherapy and individualized targeted therapy can be used in combination. Under the effect of chemoradiotherapy, some tumor cells and TME are destroyed, which is conducive to the infiltration of immune cells such as APC and CD8^+^T. The primary drug resistance (especially for desert tumors) can promote the presentation of tumor‐specific antigen, activate the release of immune killer cells and related factors, and chemotactic the infiltration of peripheral active immune cells. Assist in the selection of agents for the treatment of this type of tumor under the ICB or based on an evidence‐based assessment protocol as a medium range of treatment; combined with the analysis of individual tumor biological characteristics, such as gene mutation site detection, epigenetic analysis, signaling pathway abnormalities, tumor metabolites and energy supply, and other targeted biomarker analysis, the rational selection of highly targeted drugs of small therapeutic range types. Combined with these three levels of treatment, a comprehensive synergistic clearing effect can be achieved on the subsets of dominant and inferior cells in the tumor, reducing or completely eliminating the residual lesions, so that the drug resistance, recurrence, or metastasis of the tumor can be fundamentally controlled.

The mechanisms and treatment strategies discussed above still need to be supported by data from subsequent laboratory and clinical studies. Due to multiple combination therapy in patients with adverse reactions to the need for close observation, how to choose reasonable drug dosage, cycle, or point in time, need according to the patient's tumor type, stage, physical condition, complications, and compliance. Or it can be adjusted according to the early treatment effect and tolerance level, estimates that are more difficult to achieve a unified standardized treatment.

## CONFLICT OF INTEREST

The authors Xiaoying Wu and Zhengyi Wang declare that they have no competing interests.

## AUTHORS’ CONTRIBUTION

All authors made substantial contributions to the manuscript and gave their approval on the final version. The present publication has not been published in this form previously and is not under consideration for publication elsewhere.

## Data Availability

All data, models, and code generated or used during the study appear in the submitted article.
